# Trends and drivers of multidrug-resistant bacteria incidence in 59 Chilean intensive care units, 2015–2024: a Bayesian hierarchical analysis

**DOI:** 10.1016/j.lana.2026.101467

**Published:** 2026-04-04

**Authors:** Maria-Paz Acuña, Patricio Ross, Gerardo Carcamo, Jose-Miguel Arancibia, Ruth Rosales, Ines Ceron, Francisco Silva, Marcela Cifuentes, Patricia Garcia, Jaime Labarca, Cristóbal Cuadrado, Kasim Allel, Grupo Colaborativo Resistencia Bacteriana, Javier Caviedes, Ismael Peso, Javier Carmona, Carlos Garcés, Freddy Roach, Pedro Usedo, Patricia Gutiérrez, Rubén Muñoz, Paula Cerda, Isabel Briceño, Elizabeth Barthel, Nicolás Valdebenito, Rodrigo Ahumada, Yaritza Delgadillo, Alejandro Joyas, Gerardo Peralta, Gonzalo Wilson, Rosa Sandoval, Rubén Muñoz, Claudia Rojas, Luis Bavestrello, Dennise Covarrubias, Sonia Correa, Mildred Lima, Andrés Cornejo, Renato Ocampo, Jorge León, Manuela Cociña, Henriette Chabouty, Ignacio Aguayo, Sergio Mella, Elizabeth Villar, Gisela Riedel, Maria Eugenia Castro, Martha Quezada, Fabiola Salgado, Nicolás Rodríguez, Pamela Toloza, Álvaro Llancaqueo, Gerardo Fernández, Christian Esveile, Vanessa Villagran, Pablo Saavedra, Daniela Guzmán, Eduardo Gallegos, Natacha Garrido, Vijna Illesca, Gonzalo Rivera, Sandra Torres, Helen Stegmaier, Yazmin Pinos, Stephania Passalacqua, Soraya Daza, María Carolina Cruz, Mario Calvo, Alberto Fica, Daniel Muñoz, María Ignacia Álvarez, María Luisa Rioseco, Sebastián Barría, Celia Retamal, Mónica Pinto, Rodrigo Muñoz, Andrea Maripani, Fernando Meza, Ariel Figueroa, Renato Carrasco, Erna Cona, Diego Saa, Margareta Mühlhauser, Pablo Valenzuela, Verónica Bustamante, Vjera Triantafilo, Christian Conde, Héctor Morales, Francisco Silva, Marcela Cifuentes, Jeannette Dabanch, Fernanda Ávila, Patricia García, Álvaro Rojas, Inés Cerón, Tomás Reyes, Patricio Ross, Waldo Gutierrez, Paulette Legarraga, Dona Benadof, Mirta Acuña, Luis Valenzuela, Alejandra Céspedes, Leonardo Chanqueo, José Miguel Arancibia, Daniela Pavéz, Ana María Álvarez, Fernando Bernal, Raúl Quintanilla, Luis Delpiano, Carlos Espinoza, Marcela Cifuentes, Bélgica Barraza, Luz María Fuenzalida, María Elvira Simian, Erika Rubilar, Nicolle Flores, María Fernanda Yarad, Gloria Marín, Victoria Moreno, Paulina Coria, Paz Tabilo, Marlon Barraza, Francisca Valdivieso, Patricia Torres, Claudio González, Fernando López, Ruth Rosales, Claudia Monterrosa, Francisco Zamora, Ignacio Silva, Gustavo Saint-Pierre, Alexis Diomedi, William Acevedo, Claudia Vera, Ernesto Paya, Rodolfo Villena, Carolina Rivacoba, Maximiliano González, Pablo Cartes, Paulina Donato, Karol Villalobos, José Morales, Martín Lasso, Pamela Rojas, José Manuel Munita, Cristian Orellana, Daniela Ramírez, Leonor Jofré, Cristian Paredes, María Paz Acuña, Loriana Castillo, Lorena Porte, José Manuel Munita, Nicolle Salazar, Rafael Araos, Beatrice Hervé, Rodrigo Blamey, Cecilia Tapia, Ignacio Rodríguez, Patricio Ross, Waldo Gutiérrez

**Affiliations:** a Department of Infectious Diseases, School of Medicine, Pontificia Universidad Catolica de Chile, Santiago, Chile; b Hospital Dr. Sotero del Rio, Santiago, Chile; c Hospital La Florida, Santiago, Chile; d Red de Salud UC-CHRISTUS, Santiago, Chile; e School of Biological Sciences, Pontificia Universidad Catolica de Chile, Santiago, Chile; f Hospital San Juan de Dios, Santiago, Chile; g Complejo Asistencial Barros Luco Trudeau, Santiago, Region Metropolitana, Chile; h Hospital Clínico Universidad de Chile, Santiago, Chile; i Departamento de Laboratorios Clínicos, Escuela de Medicina, Facultad de Medicina, Pontificia Universidad Católica de Chile, Santiago, Chile; j Escuela de Salud Publica, Universidad de Chile, Santiago, Chile; k Nuffield Department of Primary Care Health Sciences, University of Oxford, Oxford, UK

**Keywords:** Intensive care unit, Multidrug resistance, Incidence, South America

## Abstract

**Background:**

Antimicrobial resistance (AMR) is a major threat in intensive care units (ICUs). Evidence on determinants of multidrug-resistant (MDR) infections in ICUs remains limited. We aimed to assess temporal, institutional, and antibiotic-use drivers of MDR incidence across 59 Chilean ICUs across 40 hospitals (2015–2024).

**Methods:**

We conducted an ecological time-trend analysis using data from the Collaborative Group on Bacterial Resistance. MDR incidence density rates (IDRs) in 1000 patient-days comprised methicillin-resistant *Staphylococcus aureus* (MRSA), vancomycin-resistant Enterococcus spp. (VRE), extended-spectrum β-lactamase (ESBL)-producing *Klebsiella pneumoniae* and *Escherichia coli*, carbapenem-resistant Enterobacterales (CRE), *Pseudomonas aeruginosa* (CRPA), *Acinetobacter baumannii* (CRAB), and carbapenemase-producing Enterobacterales (CPE). IDRs were modelled using three-level Bayesian hierarchical regressions, accounting for repeated annual measures within hospital–pathogen pairs and differences between hospitals. Models included hospital infrastructure, infectious disease specialist hours, antimicrobial stewardship (AMS) programmes, socioeconomic variables, and antibiotic use (cephalosporins, quinolones, carbapenems; in DDDs/1000 bed-days).

**Findings:**

Between 2015 and 2024, MDR incidence declined by 21% (1.82–1.44 per 1000 patient-days), driven by reductions in CRPA (4.8–1.9), MRSA (3.2–1.0), and VRE (1.4–0.9). CRE declined modestly (2.7–1.7), while CPE increased from 0 to 1.3 after 2017. Adult ICUs and public hospitals had higher IDRs than paediatric and private units. In adjusted models, quinolone use was associated with higher MDR incidence (β = 0.08, 95% CI 0.03–0.14; p = 0.004), as was carbapenem use (β = 0.06, 0.03–0.09; p < 0.0001). Each additional hour of infectious disease specialist coverage per 100 bed-days reduced MDR incidence by ∼2% (β = −0.02, −0.03 to −0.01; p = 0.023). MRSA increased with quinolones, while CRE and CRPA increased with carbapenems.

**Interpretation:**

MDR incidence in Chilean ICUs remains high and driven by quinolone and carbapenem use. Strengthening AMS and specialist oversight, alongside stricter prescribing, could reduce burdens.

**Funding:**

No funding.

## Abstract (es)

**Background:**

La resistencia a los antimicrobianos (RAM) representa una amenaza importante en las unidades de cuidados intensivos (UCI), donde convergen el uso de antibióticos de amplio espectro y la gravedad de las infecciones. En Chile, la evidencia sobre los determinantes de infecciones por microorganismos multirresistentes (MDR) en UCI sigue siendo limitada. Evaluamos los determinantes temporales, institucionales y relacionados con el uso de antibióticos de la incidencia de infecciones MDR en 59 UCI de 40 hospitales chilenos entre 2015 y 2024.

**Métodos:**

Realizamos un análisis ecológico de tendencias temporales utilizando datos del Grupo Colaborativo en Resistencia Bacteriana. Las tasas de densidad de incidencia de MDR (IDR) por 1.000 días-paciente incluyeron *Staphylococcus aureus*resistente a meticilina (MRSA), *Enterococcus spp*. resistente a vancomicina (VRE), *Klebsiella pneumoniae* y *Escherichia coli* productoras de β-lactamasas de espectro extendido (ESBL), Enterobacterales resistentes a carbapenémicos (CRE), *Pseudomonas aeruginosa* resistente a carbapenémicos (CRPA), *Acinetobacter baumannii* resistente a carbapenémicos (CRAB) y Enterobacterales productores de carbapenemasas (CPE). Las IDR se modelaron utilizando regresiones jerárquicas bayesianas de tres niveles, con medidas anuales repetidas anidadas dentro de pares hospital–patógeno, variación hospital–patógeno y diferencias entre hospitales. Los modelos incorporaron infraestructura hospitalaria, horas de especialistas en enfermedades infecciosas, presencia de programas de optimización del uso de antimicrobianos (AMS), variables socioeconómicas y uso de antibióticos (cefalosporinas, quinolonas, carbapenémicos en DDD/1.000 días-cama).

**Resultados:**

Entre 2015 y 2024, la incidencia global de MDR disminuyó en un 21%, de 1.82 a 1.44 infecciones por 1.000 días-paciente, impulsada principalmente por reducciones en CRPA (4.8 a 1.9), MRSA (3.2 a 1.0) y VRE (1.4 a 0.9). CRE (2.7 a 1.7) mostró disminuciones menores, mientras que CPE aumentó marcadamente de 0 a 1.3 por 1.000 días-paciente después de 2017. Las UCI de adultos y los hospitales públicos presentaron IDR más altas que las unidades pediátricas y privadas. En los modelos completamente ajustados, el uso de quinolonas se mantuvo positivamente asociado con la incidencia global de MDR (β = 0.08; IC 95% 0.03–0.14; p = 0.004), mientras que el uso de carbapenémicos también incrementó el riesgo (β = 0.06; IC 95% 0.03–0.09; p < 0.0001). Cada hora adicional de cobertura de especialistas en enfermedades infecciosas por 100 días-cama redujo la incidencia de MDR en aproximadamente un 2% (β = −0.02; IC 95%−0.03 a −0.01; p = 0.023). Los análisis específicos por combinación fármaco–patógeno mostraron que la incidencia de MRSA aumentó con el uso de quinolonas (β = 0.29; IC 95% 0.14–0.44; p < 0.0001), mientras que CRE (β = 0.11; IC 95% 0.05–0.16; p < 0.0001) y CRPA (β = 0.09; IC 95% 0.01–0.17; p = 0.032) aumentaron con el uso de carbapenémicos.

**Interpretación:**

La incidencia de MDR en las UCI chilenas sigue siendo alta en comparación con entornos de altos ingresos y está asociada con el uso de quinolonas y carbapenémicos. El fortalecimiento de la cobertura de AMS, particularmente en hospitales públicos, y la garantía de una adecuada supervisión por especialistas en enfermedades infecciosas podrían mitigar sustancialmente la transmisión de MDR. Se requieren guías de prescripción revisadas para limitar la exposición innecesaria a carbapenémicos y quinolonas, con el fin de alinear la práctica clínica con los patrones de resistencia en evolución.

**Research in context**

**Evidence before this study**

We searched PubMed and Scopus (January 1, 2010–September 30, 2025) using the MeSH terms related to (“intensive care unit”) AND (“antimicrobial resistance”) AND (“antibiotic use”) AND (“Latin America” OR “Chile”) including all 2024 critical and high-priority antimicrobial-resistant (AMR) pathogens, as defined by the WHO. We found multicentre studies describing AMR incidence in intensive care units (ICUs), but none that jointly analysed antibiotic use (ABU), institutional, and socioeconomic drivers using longitudinal data. Previous work in Chile reported rising extended-spectrum β-lactamase (ESBL) and carbapenem-resistant infections and a 1.4-fold excess mortality among AMR bloodstream infections, yet these studies either aggregated across wards or excluded antibiotic exposure data. No study to date has applied quantified antibiotic-specific and institutional determinants of MDR infection incidence across ICUs in Latin America.

**Added value of this study**

This is the first national, multicentre analysis to quantify the relationship between antibiotic use, institutional capacity, and multidrug-resistant (MDR) infection incidence in Latin America, analysing 59 ICUs in 40 Chilean hospitals between 2015 and 2024. Using three level Bayesian hierarchical models, we disentangled temporal, hospital-level, and bacterial level variation in incidence density rates. We show that quinolone and carbapenem use were the strongest antibiotic-specific predictors of MDR infections, particularly methicillin resistant *Staphylococcus aureus* (MRSA) with quinolones and carbapenem-resistant Enterobacterales (CRE) and *Pseudomonas aeruginosa* (CRPA) with carbapenems. Greater infectious disease specialist availability was independently associated with lower MDR incidence. These results provide the first robust quantification of antibiotic- and institution specific drivers of ICU-level AMR in Latin American settings.

**Implications of all the available evidence**

Our findings highlight modifiable institutional and prescribing factors driving ICU-level MDR in Chile and similar health systems. Reducing quinolone and carbapenem use, expanding infectious disease specialist coverage, and ensuring full implementation of antimicrobial stewardship programmes in public hospitals could markedly reduce MDR incidence. The study establishes a scalable modelling framework integrating antibiotic consumption, hospital infrastructure, and local socioeconomic context to guide data-driven stewardship policies, consistent with WHO's Global AMR Surveillance System (GLASS) and Sustainable Development Goal 3.d on strengthening health system resilience to AMR threats.

## Introduction

Antimicrobial resistance (AMR) poses severe health and economic challenges, with an estimated 8.22 million (6.85–9.65) associated deaths projected by 2050.^1^ Antibiotic use (ABU), the primary driver,^2^ rose by 11% from 13.7 in 2016 to 15.2 daily defined doses (DDDs) per 1000 inhabitants per day in 2023.^3^ Projections estimated a 52.3% increase, reaching 75 billion DDDs by 2030. The growing antibiotic use (ABU) of broad-spectrum antibiotics has intensified selective pressure, driving complex resistance mechanisms (e.g., carbapenemase production) and multi-drug resistance (MDR).^4^ This is particularly critical in intensive care units (ICUs), which present the highest incidence of MDR bacteria, the most clinically severe infections, increased healthcare costs, and the greatest overall ABU.^5^, ^6^, ^7^

In Chile, as observed in other countries in the region,^8^ AMR is a growing concern. For example, a recent study found that AMR produced 1.44 (1.22–1.71) greater in-hospital mortality in patients with bacteraemia,^9^ compared to susceptible bacteria, based on all drug-bug combinations classified as high- or critical priority in the WHO pathogen list.^10^ CRE and MRSA, often classified as MDR, showed the highest likelihood of ICU admissions (odds ratio ‘OR’ = 1.44 and 1.59, respectively) and excess economic costs ($12,233 and $15,970, respectively), compared to their susceptible counterparts.^9^ However, multicentre studies exclusively focussing on ICUs, without mixing wards, remain scarce. One of the most comprehensive analyses, conducted across 20 Chilean ICUs (2014–2015), found that most prevalent pathogens were ESBL-producing *Klebsiella pneumoniae* [4.72/1000 bed-days (1.21–13.89)] and MRSA [3.85 (0.71–12.66)].^11^ This study; however, focused solely on incidence, excluding ABU, which may have overlooked critical drivers behind these estimates. Other studies in Chile have found increasing ABU over time,^12^ especially after the COVID-19 pandemic and particularly among carbapenems, colistin and broad-spectrum antibiotics,^13^ which might have shifted local epidemiology.

The Collaborative Group on Bacterial Resistance (GCRB) has been instrumental in monitoring AMR trends and ABU in Chile,^11^^,^^14^ comprising 57 hospitals, serving both paediatric and adult populations of varying complexities. Factors such as poverty, overcrowding, limited access to sanitation, inconsistent healthcare access and hospital complexity are known to exacerbate the spread of AMR rates between 2008 and 2017 in Chilean hospitals as a whole.^14^ Despite these efforts, data on socioeconomic and demographic drivers of MDR incidence in ICUs remain scarce. Understanding these local determinants, alongside clinical and institutional factors, and previously overlooked ABU,^14^ is essential for disentangling unbiased spatiotemporal relationships, particularly in the context of pre- and post-COVID-19 trends.

This study focuses on identifying clinical, institutional, and socioeconomic risk factors associated with the incidence of MDR bacteria across the critical drug-bug combinations in 59 ICUs among 40 hospitals across Chile (2015–2024).

## Methods

### Study design and population

We conducted an ecological time-trend study of ICUs in 40 Chilean hospital-level health centres from 2015 to 2024. We included 59 ICUs, among which 37 corresponded to adult and 22 to paediatric ICUs, with some from same hospital venue. We included participating hospitals from the Collaborative Group on Bacterial Resistance of the Chilean Society of Infectious Diseases. A full list of hospitals-years is available in Supplementary Tables S1 and S2.

### Incidence density rate (IDR)

We calculated the IDR as the number of pathogen-specific new infections per 1000 patient-days for paediatric and adult ICUs. All ICU clinical isolates were included in the incidence analysis, and resistance breakpoints were determined according to the Clinical and Laboratory Standards Institute ‘CLSI’ guidelines, as applicable to the year of report.^15^ More details on inclusion and exclusion criteria are provided in Supplementary Text S1. We followed the formula [(number of isolates/accumulated length of hospital stay in the ICU)∗1000]. We only considered those pathogens defined as high- or critical-priority by the WHO,^10^ which included methicillin-resistant *S. aureus* (MRSA), vancomycin-resistant Enterococcus species (VRE), Extended-spectrum β-lactamase *K. pneumoniae* (ESBL KP) and *Escherichia coli* (ESBL EC), carbapenem-resistant Enterobacterales (CRE), carbapenemase-producing Enterobacterales (CPE), carbapenem-resistant *P. aeruginosa* (CRPA), and carbapenem-resistant *Acinetobacter baumannii* (CRAB). The crude number of isolates over time, stratified by pathogen–antibiotic combination, is presented in Supplementary Table S3.

### Independent variables

We categorised the independent variables into three groups. The first group focused on hospital infrastructure. Hospital type (public or private) and total bed capacity (number) were retrieved from the Department of Statistics and Health Information (DEIS).^16^ We collated the number of ICU beds, availability of cardiac surgery services (yes/no), weekly hours per 100 beds dedicated by infectious diseases specialists, provision of treatment for haematological neoplasms (yes/no), neurosurgical disorders (yes/no), and the implementation of an antimicrobial stewardship program (yes/no) by asking hospitals directly. The second group encompassed hospital-reported ABU, measured as the daily defined doses (DDD) of third- and fourth-generation cephalosporins, quinolones, and carbapenems per 100 ICU bed-days. DDDs were converted using WHO-ATC conversions.^17^ The third group comprised sociodemographic characteristics of the populations served by healthcare institutions, most of which were derived from the National Socioeconomic Characterization Survey (CASEN).^18^ We used population weights at the municipality level to better characterise hospital catchment areas. Since CASEN is conducted every two years, data for the missing years were interpolated. Variables included the percentage of individuals aged ≥60 years, the percentage of the population in rural areas, and rates of poverty, extreme poverty, and multidimensional poverty. Additional factors were overcrowding, the proportion of non-Chilean individuals assigned to the healthcare facility, and access to basic services such as water, sanitation, and hygiene (WASH).

All variables, including their definitions, calculation formulae, and data sources, are detailed in Supplementary Tables S4–S7. We followed the Strengthening the reporting of observational studies in epidemiology (STROBE) guidelines.

### Statistical analyses

We employed descriptive analyses computing medians and interquartile ranges (IQR) used for continuous variables, while proportions were presented for categorical variables. We computed regional averages of IDR and ABU from data aggregated across the observed hospitals and generated corresponding heatmaps. We fitted a three-level Bayesian hierarchical model to estimate temporal and contextual determinants of IDRs across bacterial species and hospitals. Level 1 represented repeated annual IDR measurements within each hospital–bacterium pair. Level 2 captured variation between hospital–bacterium combinations through random intercepts, and Level 3 accounted for broader between- and within-hospital differences including adults and paediatric ICUs. See Supplementary Table S8 and Supplementary Figure S1 for the model and hierarchical structure. IDRs were modelled using a Gaussian likelihood, with time and ICU, hospital type, infectious disease bed capacity, poverty, and migration as fixed effects in progressively adjusted models. Weakly informative priors (*Normal* [0.5] for fixed effects; *Exponential* [1] for random effect standard deviations) were used to regularise estimation. Four MCMC chains of 4000 iterations (2000 warm-up) were run per model. Convergence was verified via R-hat (<1.01), effective sample size (>400), and visual trace inspection. Posterior summaries are reported as means and 95% credible intervals (CIs), with probabilities of direction (p-value) used to quantify evidence of association. Bayesian hierarchical models were selected to account for data clustering and repeated measures, conditions under which standard regression assumptions of independence are violated. Bayesian hierarchical models also handle unbalanced datasets and missing data assumed to be missing at random (MAR).

We followed a five-tier modelling approach. First, a causal directed acyclic graph (DAG) constructed using DAGitty v2.3 guided variable selection to avoid collider bias (Supplementary Figure S2). Second, only the most relevant variables were included as independent predictors, selected based on variable importance identified through preliminary LASSO regressions. Third, we sequentially added independent predictors, including paediatric ICU, hospital type, infectious diseases specialist hours per 100 bed-days, and poverty in the ICU catchment area. Fourth, we assessed the impact of antibiotic use—cephalosporins, carbapenems, and quinolones—expressed as DDDs per 1000 patient-bed days, both individually and jointly, in unadjusted models and in models adjusted for the variables specified in the third step. Fifth, we estimated subgroup hierarchical regressions by stratifying the sample by bacteria–drug combination, reducing the model hierarchy to two levels by omitting the bacteria–hospital layer.

We computed the Intraclass Correlation Coefficients (ICCs) to evaluate the proportion of total variance explained by hierarchical levels. Model's goodness-of-fit were evaluated with the Akaike Information Criterion (AIC), and all analyses were conducted using R (version 4.5.3), mainly using the ‘*brms*’ package.

### Ethical approval

Not required, as only publicly available data were used.

### Role of the funding source

The funder of the study had no role in study design, data collection, data analysis, data interpretation, or writing of the report.

## Results

### Descriptive statistics of the included ICUs

Among 59 ICUs included from 2015 to 2024, the median participation duration was 3 years (IQR 3–4) (Table 1) although the number of contributing ICUs varied by year (Supplementary Table S9). Median ICU capacity was 15 beds (IQR 8–28), with hospitals averaging 487 total beds (IQR 367–558). Median infectious disease specialist availability was 14.3 weekly hours per 100 bed-days (IQR 8.7–24.1). Antimicrobial stewardship programmes were in place in 59% of ICU–time combinations (n = 665 hospital–time points). Observations from private hospitals were limited (19%, n = 359), and 43% of observations were from hospitals in Santiago (n = 790). Median overall MDR infection incidence was 1.44 per 1000 patient bed-days (IQR 0.35–3.50), highest for ESBL-producing *K. pneumoniae* (3.78, IQR 1.9–6.76) and CRE (2.06, IQR 0.73–3.98). Median ABU was 24.1 DDD per 1000 bed-days for cephalosporins, 5.2 for quinolones, and 18.2 for carbapenems. Populations served by these hospitals had a median of 21% aged ≥60 years, 7% living in poverty, and 23% in multidimensional poverty (five-dimension measure), with 10% in overcrowded housing and 3% lacking basic sanitation. As shown in Table 1, adults and paediatrics displayed differences. Adult ICUs had greater carbapenem (19.4 *vs.* 5.3 DDDs) and quinolone use (5.5 *vs.* 2.0 DDDs) and higher ESBL *K. pneumoniae* (4.7 *vs.* 2.2) and MRSA (2.6 *vs*. 0.5) incidences per 1000 patient-bed days. Paediatric ICUs served more socioeconomically deprived populations.

**Table 1:** Descriptive profile of the included ICU, Chile, 2015–2024.

Variable	Adults	Paediatric	Total
**ICU characteristics**			
Number of participating ICUs (n)	37	22	59
Average duration of participation, expressed in years (mean, IQR)	3 (3–4)	3 (3–4)	3 (3–4)
Total number of hospital beds beyond ICU (median, IQR)	443 (362–550)	503.5 (391–572.75)	487 (367–558)
Number of ICU beds (median, IQR)	16 (11–31.5)	11 (7–19)	15 (8–28)
Infectious diseases specialist hours/100 bed-days (median, IQR)	18.07 (12.27–24.05)	9.93 (3.12–24)	14.27 (8.73–24.05)
Observations from hospitals with antimicrobial stewardship in place (n, %)	448 (62%)	217 (54%)	665 (59%)
Observations from private hospital (n, %)	312 (27%)	47 (7%)	359 (19%)
Observations from hospitals based in the capital city (n, %)	531 (45%)	259 (38%)	790 (43%)
**Incidence of MDR ×1000 patient bed-days in ICUs (median, IQR)**			
CPE	0.26 (0–1.29)	0 (0–0.20)	0.06 (0–0.86)
CRAB	0.36 (0–1.17)	0 (0–0.07)	0 (0–0.85)
CRE	3.4 (1.57–5.4)	0.94 (0.2–1.98)	2.06 (0.73–3.98)
CRPA	3.26 (1.93–5.34)	1.39 (0.51–2.69)	2.51 (1.21–4.78)
ESBL *E. coli*	2.03 (1.32–3.45)	0.81 (0.31–1.64)	1.67 (0.85–2.82)
ESBL *K. pneumoniae*	4.69 (2.66–7.35)	2.22 (0.74–4.43)	3.78 (1.9–6.76)
MRSA	2.57 (1.1–5.17)	0.54 (0.15–1.22)	1.61 (0.56–3.88)
VRE	1.59 (0.83–2.52)	0.1 (0–0.53)	0.97 (0.24–1.96)
Total incidence for all pathogen-drug combinations	2.15 (0.85–4.24)	0.53 (0–1.64)	1.44 (0.35–3.5)
**Antibiotic use in ICUs (DDDs per 1000 hospital-bed days) (median, IQR)**			
Cephalosporins	25.24 (18.59–31.34)	18.07 (14.3–24.57)	24.12 (17.79–30.59)
Quinolones	5.50 (3.37–8.98)	2.00 (1.15–4.07)	5.22 (3.08–7.88)
Carbapenems	19.41 (14.77–27.13)	5.31 (3.69–10.1)	18.19 (11.28–25.27)
**Sociodemographic variables of the beneficiary population (%, IQR)**			
Aged 60 years or older^a^	21.20 [16.27, 23.82]	20.99 [18.73, 22.95]	21.20 [18.01, 23.59]
Population living in rural areas	6.69 [3.70, 16.63]	8.27 [3.70, 25.20]	7.05 [3.70, 24.4]
Migrant population	2.64 [0.99, 5.43]	2.68 [1.60, 6.34]	2.64 [1.20, 5.44]
Living in income-based poverty	6.57 [4.42, 10.66]	7.83 [5.024, 11.955]	7.02 [4.42, 11]
Living in extreme income-based poverty	1.85 [0.43, 3.42]	2.89 [1.515, 3.768]	2.71 [0.68, 3.57]
Living in multidimensional poverty (4 dimensions)	18.72 [8.67, 23.02]	22.23 [18.72, 23.66]	20.65 [14.73, 23.12]
Living in multidimensional poverty (5 dimensions)	20.77 [10.32, 25.33]	24.79 [20.77, 26.14]	23.03 [15.96, 25.84]
Living in overcrowded conditions	8.93 [6.80, 12.63]	12.07 [8.93, 13.98]	9.85 [7.48, 13.16]
Deficient sanitation (limited access to basic services)	3.13 [1.34, 6.34]	4.33 [1.98, 9.33]	3.27 [1.70, 7.29]

Notes: ICU, Intensive care unit; IQR, Interquartile range (25th percentile, 75th percentile). DDD, Daily defined doses. We removed 2019 and 2020 because of misreporting due to the COVID-19 pandemic. CPE, Carbapenemase-producing Enterobacterales; CRAB, Carbapenem-resistant *Acinetobacter baumannii*; CRE, Carbapenem-resistant Enterobacterales; CRPA, Carbapenem-resistant *Pseudomonas aeruginosa*; ESBL ECO, Extended spectrum beta-lactamase producing *Escherichia coli*; ESBL KPN, Extended spectrum beta-lactamase producing *Klebsiella pneumoniae;* MRSA, Methicillin-resistant *Staphylococcus aureus;* VRE, Vancomycin-resistant *Enterococcus* species.

a This variable means the percentage of the population assigned to the health institution aged 60 years or older.

### Variability in IDRs and ABU over time

**Fig. 1:**
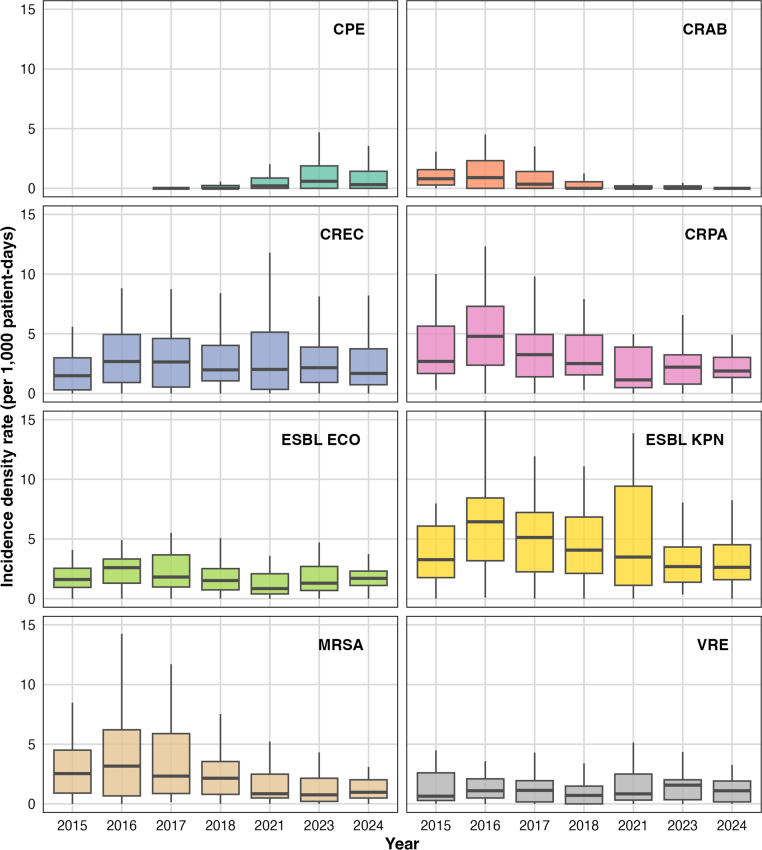
Temporal incidence trends of major antimicrobial-resistant pathogens in Chilean ICUs, 2015–2024. Notes: ICU, Intensive care unit. We removed 2019 and 2020 because of misreporting due to the COVID-19 pandemic. CPE, Carbapenemase-producing Enterobacterales; CRAB, Carbapenem-resistant *Acinetobacter baumannii*; CREC, Carbapenem-resistant Enterobacterales; CRPA, Carbapenem-resistant *Pseudomonas aeruginosa*; ESBL ECO, Extended spectrum beta-lactamase producing *Escherichia coli*; ESBL KPN, Extended spectrum beta-lactamase producing *Klebsiella pneumoniae*; MRSA, Methicillin-resistant *Staphylococcus aureus*; VRE, Vancomycin-resistant Enterococcus species.

**Fig. 2:**
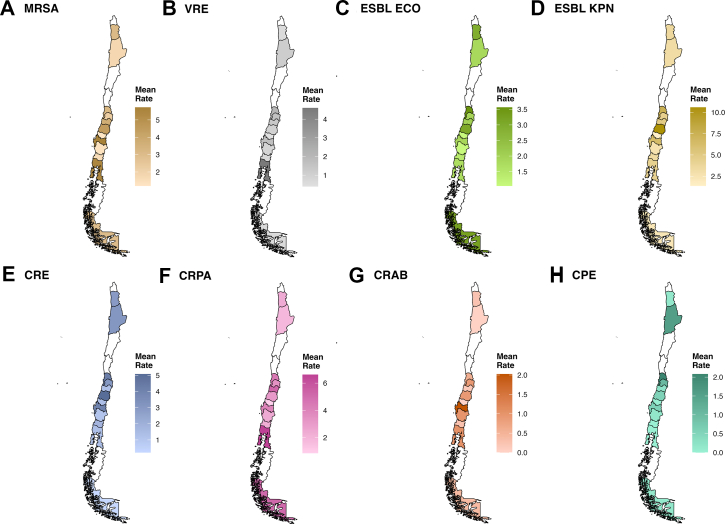
Average incidence density rate per 1000 patient-days of major antimicrobial-resistant pathogens in Chilean ICUs across the 2015–2024 time period. Notes: The average resistance rate is presented in maps using the mean rate of the incidence density rate per 1000 patient-days. The figure presents aggregated AMR rates across the study period. White areas (regions) indicate no hospital data available. ICU, Intensive care unit. We removed 2019 and 2020 because of misreporting due to the COVID-19 pandemic. CPE, Carbapenemase-producing Enterobacterales; CRAB, Carbapenem-resistant *Acinetobacter baumannii*; CRE, Carbapenem-resistant Enterobacterales; CRPA, Carbapenem-resistant *Pseudomonas aeruginosa*; ESBL ECO, Extended spectrum beta-lactamase producing *Escherichia coli*; ESBL KPN, Extended spectrum beta-lactamase producing *Klebsiella pneumoniae*; MRSA, Methicillin-resistant *Staphylococcus aureus*; VRE, Vancomycin-resistant Enterococcus species.

Across 2015–2024, incidence of MDR pathogens declined overall but remained heterogeneous across species (Fig. 1). ESBL-producing *K. pneumoniae* showed the highest rates, with median incidence peaking at 6.4 (IQR 3.2–8.4) per 1000 patient-days in 2016 and falling to 2.6 (IQR 1.6–4.5) by 2024. CRE and CRPA followed, decreasing from 2.7 (IQR 0.9–4.9) and 4.8 (IQR 2.4–7.3) to 1.7 (IQR 0.7–3.7) and 1.9 (IQR 1.3–3.0), respectively. ESBL-producing *E. coli* remained moderately frequent, at 1.6 (IQR 0.9–2.6) in 2015 and 1.7 (IQR 1.1–2.3) in 2024. MRSA incidence decreased from 3.2 (IQR 0.7–6.2) to 1.0 (IQR 0.5–2.0), and VRE stabilised around 1.1 (IQR 0.2–1.9). CPE and CRAB remained uncommon throughout, but CPE rose quickly from 0 to 1.27 between 2017 and 2024. Geographically and over time, ESBL-*K. pneumoniae* exhibited the highest mean IDR (up to 10 per 1000 patient-days) concentrated in the central region, similar to peaks for MRSA (≥5 in the central–southern area) and CRPA (≥6 in the south) (Fig. 2).

**Fig. 3:**
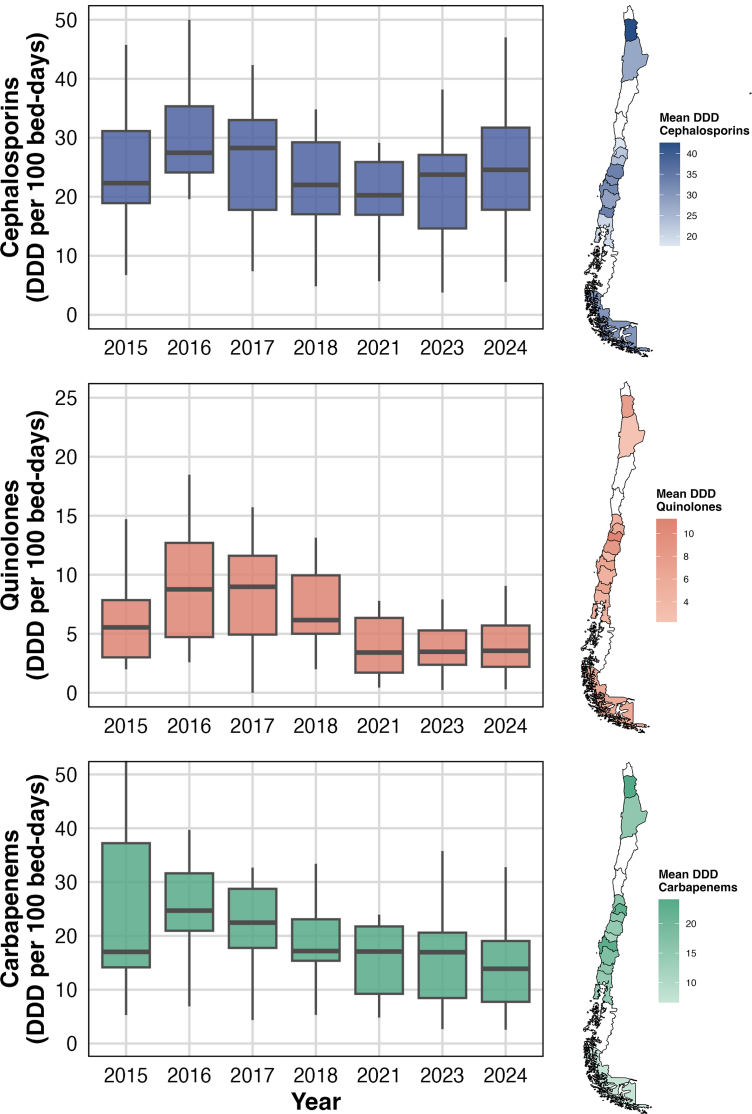
Observed antibiotic use of cephalosporins, quinolones, and carbapenems in Chilean ICUs, 2015–2024. Notes: DDD, Daily defined doses; ICU, Intensive care unit. We removed 2019 and 2020 because of misreporting due to the COVID-19 pandemic.

Fig. 3 shows trends in ABU over time across observed ICUs. Overall cephalosporin use increased from a median of 22.3 (IQR 18.9–31.1) DDDs per 100 bed-days in 2015 to 24.6 (IQR 17.8–31.7) in 2024, peaking in 2017 (28.3, IQR 17.8–33.0). Overall quinolone use dropped from 5.5 (IQR 3.0–7.9) to 3.6 (IQR 2.2–5.7) over the same period, with highest use in 2017 (median 8.9, IQR 4.9–11.6). Carbapenem use declined from 17.0 (IQR 14.2–37.2) to 13.9 (IQR 7.7–19.0), following a greatest point in 2016 (24.7 [IQR 21.0–31.6]). Spatially, ABU peaked in the northernmost region (i.e., Tarapacá) showing the highest intensities, exceeding 40 DDDs for cephalosporins, 10 for quinolones, and 20 for carbapenems.

Adult ICUs showed greater IDRs across all pathogens, particularly CRAB, CRE, and ESBL producers, while paediatric ICUs had lower and more stable rates over time (Supplementary Figures S3 and S4), with spatial heterogeneities across regions (Supplementary Figures S5 and S6). Public hospitals showed higher and more variable incidence rates, especially for CRAB, MRSA, VRE and ESBL pathogens (Supplementary Figures S7 and S8).

### Sociodemographic and hospital determinants of MDR IDRs

Models 0–4 (Table 2) showed consistent temporal declines in MDR incidence (β ≈ −0.13 to −0.25, p < 0.0001) and persistent differences by ICU type. In the fully adjusted model (Model 5), incidence fell over time (β = −0.21, 95% confidence intervals ‘CI’ −0.35, −0.06; p 0.003), with markedly lower rates in paediatric ICUs (β = −2.43, 95% CI −2.77, −2.09; p < 0.0001) and private hospitals (β = −0.76, 95% CI −1.32, −0.19; p = 0.009). While poverty and migrant share did not show significant association, infectious diseases specialist hours were protective (β = −0.02, 95% CI −0.03, −0.01; p = 0.023). Model 5 achieved the best fit (R^2^_marginal_ = 0.16; R^2^_conditional_ = 0.54; ICC = 0.42), indicating that fixed effects explained 16% of MDR variance, the full model (including random effects) explained 54%, and 42% of total variance was attributable to between-hospital and hospital–bacteria level clustering differences.

**Table 2:** Results from Bayesian hierarchical models including sociodemographic and hospital variables for the incidence rate per 1000 patient-days of multidrug-resistant infections in ICU settings, 2015–2024.

Variable	Model 0 Coeff. [95% CI]	p	Model 1 Coeff. [95% CI]	p	Model 2 Coeff. [95% CI]	p	Model 3 Coeff. [95% CI]	p	Model 4 Coeff. [95% CI]	p	Model 5 Coeff. [95% CI]
Intercept	2.89 [2.43, 3.34]	<0.0001	3.31 [2.87, 3.75]	<0.0001	3.55 [3.04, 4.03]	<0.0001	4.54 [3.98, 5.10]	<0.0001	4.17 [3.24, 5.10]	<0.0001	4.20 [3.24, 5.17]
Time [year]	−0.15 [–0.21, −0.08]	<0.0001	−0.13 [–0.19, −0.06]	<0.0001	−0.13 [–0.19, −0.07]	<0.0001	−0.25 [–0.37, −0.13]	<0.0001	−0.21 [–0.35, −0.07]	0.004	−0.21 [–0.35, −0.06]
Paediatric ICU			−1.60 [–1.88, −1.33]	<0.0001	−1.64 [–1.91, −1.36]	<0.0001	−2.45 [–2.79, −2.13]	<0.0001	−2.44 [–2.77, −2.11]	<0.0001	−2.43 [–2.77, −2.09]
Private hospital					−0.57 [–1.13, −0.01]	0.048	−0.88 [–1.36, −0.42]	<0.0001	−0.74 [–1.28, −0.21]	0.007	−0.76 [–1.32, −0.19]
Infectious diseases specialist hours/100 bed-days							−0.02 [–0.03, −0.01]	0.009	−0.02 [–0.03, −0.00]	0.028	−0.02 [–0.03, −0.00]
Poverty									2.58 [–2.50, 7.76]	0.33	2.43 [–2.82, 7.59]
**Random effects**											
σ^2^	6.5		6.03		6.03		4.36		4.36		4.36
τ_00_ (hospital-bacteria)	2.24		2.32		2.31		3.3		3.3		3.3
τ_00_ (hospital)	1.22		0.94		0.86		0.25		0.24		0.24
ICC	0.23		0.25		0.25		0.42		0.42		0.42
R^2^ marginal/conditional	0.008/0.354		0.068/0.401		0.070/0.402		0.156/0.541		0.158/0.541		0.158/0.541
Observations	All models had a total of1852 observations from 40 ICU settings comprising 317 hospital–bacteria combinations										

Notes: ICC, Intraclass correlation; ICU, Intensive care unit; Coeff, Coefficient; CI, Confidence intervals.

### Antibiotic use as determinant of MDR and drug-bug combination IDRs

Models 1–3 (Table 3) showed that increasing ABU was positively associated with MDR incidence. Cephalosporin use showed a significant association early on (Model 1, β = 0.04, 95% CI 0.02–0.06; p < 0.0001; meaning that each additional DDD is associated with an increase of 0.04 MDR cases per 1000 bed-days) but disappeared after adjustment for fixed hospital-level covariates (Model 4). In contrast, quinolone (β = 0.11, 95% CI 0.05–0.16; p < 0.0001) and carbapenem use (β = 0.05–0.06; 95% CI 0.03–0.09; p < 0.0001) remained consistently associated with MDR infection incidence across adjusted models. In the fully adjusted specification (Model 7), which incorporated all antibiotic classes and contextual covariates, both quinolone (β = 0.08, 95% CI 0.03–0.14; p = 0.004) and carbapenem use (β = 0.06, 95% CI 0.03–0.09; p < 0.0001) retained significant positive associations with MDR incidence, but cephalosporins did not (Supplementary Figure S9). As for Model 7, fixed effects explained 14% of the total variance, the full model 67%, and 60% of total variability (i.e., ICC) was attributable to between-hospital and hospital–bacteria clustering.

**Table 3:** Results from Bayesian hierarchical models assessing antibiotic use as the main determinant of multidrug resistance incidence rate per 1000 patient-days in ICU settings, 2015–2024.

Variable	Model 1 Coeff. [95% CI]	p	Model 2 Coeff. [95% CI]	p	Model 3 Coeff. [95% CI]	p	Model 4 Coeff. [95% CI]	*p*	Model 5 Coeff. [95% CI]	p	Model 6 Coeff. [95% CI]	p	Model 7 Coeff. [95% CI]	p
Intercept	2.48 [1.58, 3.38]	<0.0001	2.02 [1.28, 2.75]	<0.0001	0.56 [−0.31, 1.42]	0.22	4.27 [2.55, 6.05]	<0.0001	4.04 [2.38, 5.63]	<0.0001	3.43 [1.92, 4.96]	<0.0001	2.24 [0.30, 4.11]	0.024
Time [year]	−0.18 [−0.27, −0.08]	<0.0001	−0.06 [−0.17, 0.05]	0.26	−0.00 [−0.10, 0.10]	0.97	−0.05 [−0.25, 0.15]	0.66	−0.07 [−0.29, 0.14]	0.52	0.01 [−0.19, 0.20]	0.95	0.09 [−0.15, 0.32]	0.46
Cephalosporins use in DDD per 1000 hospital bed-days	0.04 [0.02, 0.06]	<0.0001					0.02 [−0.01, 0.06]	0.15					0.01 [−0.02, 0.04]	0.52
Quinolones use in DDD per 1000 hospital bed-days			0.17 [0.12, 0.22]	<0.0001					0.11 [0.05, 0.16]	<0.0001			0.08 [0.03, 0.14]	0.004
Carbapenem use in DDD per 1000 hospital bed-days					0.11 [0.09, 0.14]	<0.001					0.05 [0.03, 0.08]	<0.0001	0.06 [0.03, 0.09]	<0.0001
FE variables^a^							✓		✓		✓		✓	
**Random effects**														
σ^2^	6.94		6.85		6.33		3.68		3.71		3.65		3.66	
τ_00_ (hospital-bacteria)	2.41		2.48		2.53		5.83		6.02		5.78		5.89	
τ_00_ (hospital)	1.51		1.14		1.48		0.77		0.53		0.21		0.2	
ICC	0.22		0.24		0.25		0.57		0.59		0.6		0.6	
R^2^ marginal/conditional	0.03/0.04		0.07/0.42		0.13/0.45		0.09/0.65		0.11/0.67		0.12/0.66		0.14/0.67	
Observations	All models had a total of 1852 observations from 59 ICU settings comprising 317 hospital–bacteria combinations													

Notes: DDD, Daily defined doses; ICC, Intraclass correlation; ICU, Intensive care unit; Coeff, Coefficient; CI, Confidence intervals; FE, Fixed effect.

a FE variables indicate fixed effect variables such as paediatric ICU, private hospital, infectious disease hours, poverty.

**Fig. 4:**
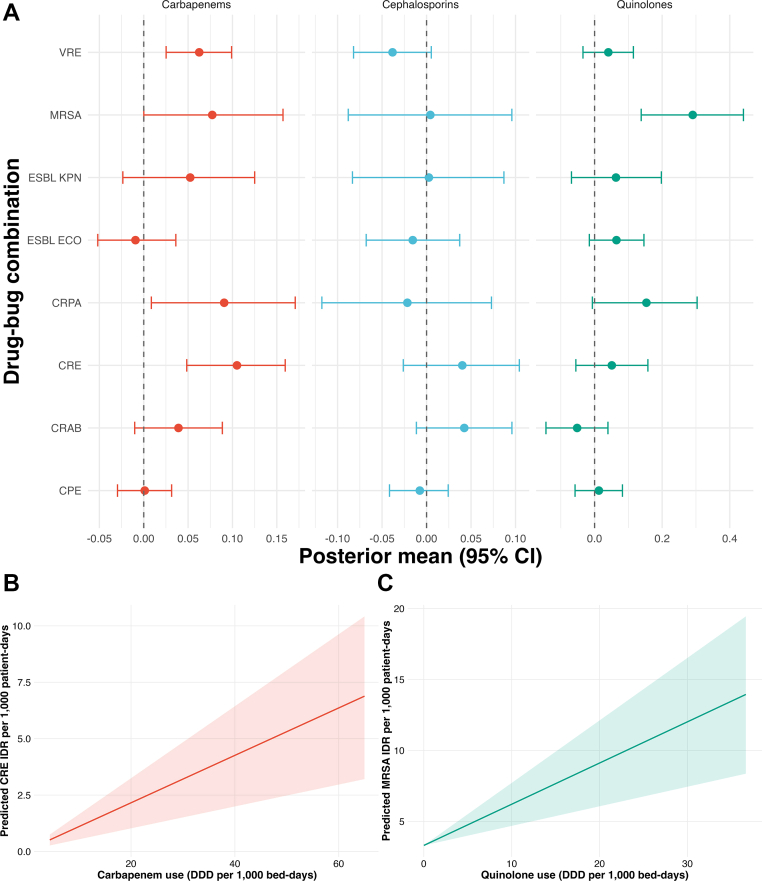
Posterior estimates from a Bayesian hierarchical model evaluating the impact of antibiotic use on the incidence rate per 1000 patient-days of multidrug-resistant infections in ICU settings, 2015–2024, after adjustment for confounders. Notes: A. Figure shows the impact of antibiotic use (carbapenem, quinolones and cephalosporins) on IDR per 1000 patient-days, by bug–drug combination. B. displays the association between carbapenem use and our predicted CRE IDR from subgroup analysis. C. displays the association between quinolone use and our predicted MRSA IDR from subgroup analysis. Full model is found in Supplementary Table S9. Predicted incidence density rate was calculated among all pathogen-antibiotic combinations, per 1000 patient-bed days. CPE, Carbapenemase-producing Enterobacterales; CRAB, Carbapenem-resistant *Acinetobacter baumannii*; CRE, Carbapenem-resistant Enterobacterales; CRPA, Carbapenem-resistant *Pseudomonas aeruginosa*; ESBL ECO, Extended spectrum beta-lactamase producing *Escherichia coli*; ESBL KPN, Extended spectrum beta-lactamase producing *Klebsiella pneumoniae*; MRSA, Methicillin-resistant *Staphylococcus aureus*; VRE, Vancomycin-resistant Enterococcus species.

In time- and antibiotic-adjusted subgroup analyses by drug–bug combination, quinolone use (β = 0.11–0.32, p < 0.014 across models) was positively associated with higher incidence of most MDR infections (Supplementary Table S8). Carbapenem use also showed consistent positive associations (β = 0.03–0.16, p < 0.032), with CRE and CRPA showing the highest (β = 0.16 and 0.14, respectively, p < 0.0001). Cephalosporin use was not significantly associated with incidence except for MRSA and CRAB. Fully adjusted hierarchical models (Fig. 4 and Supplementary Table S11) confirmed that carbapenem use remained significantly associated with higher incidence of CRE (β = 0.11, 95% CI 0.05–0.16, p < 0.0001; Fig. 4B) and CRPA (β = 0.09, 95% CI 0.01–0.17, p = 0.032). Conversely, MRSA incidence increased with quinolone use (β = 0.29, 95% CI 0.14–0.44, p < 0.0001; Fig. 4A and C), whereas other pathogens were mostly unaffected.

## Discussion

This study provides a comprehensive analysis of MDR incidence in Chilean ICUs, highlighting significant burdens in both adult and paediatric populations for most critical pathogens following the WHO's pathogen priority list. Our findings reveal substantial heterogeneity in MDR incidence, driven by ABU and institutional and socioeconomic factors shaping resistance dynamics.

The IDRs observed in this study were greater than those previously reported among HICs.^19^, ^20^, ^21^ For instance, MRSA incidence in our study was almost four times higher than rates reported among 354 and 75 ICUs in Germany and Italy, respectively, with 0.6 per 1000 patient-days in those studies.^19^^,^^21^ A French multicentre study reported incidence densities of 1.14 MRSA and 1.63 ESBL-Enterobacterales per 1000 patient-days among 933 hospitals: 52% and 74% lower than ours, respectively, dominated by *E. coli*.^20^ In contrast, an Italian study reported 3.0 CRAB and 2.3 ESBL-*K. pneumoniae* per 1000 patient-days.^21^ These differences likely reflect gaps in infection control and antimicrobial stewardship implementation compared with European settings, where coordinated national programmes were established earlier.^22^ For instance, Chile's national hospital-based AMS programme (PROA) became mandatory in December 2020 (Decree N°210),^23^ but full implementation was delayed until 2024, due to the COVID-19 pandemic. Despite this, we observed a 21% reduction in overall MDR incidence over 10 years, probably driven by MRSA and VRE reduction, as reported by the CDC.^24^ In addition, parallel national policies, including enhanced standards for infection prevention and control (Technical Standard No. 199, 2018), the National AMR Plan (since 2017), and Supreme Decree No. 7 (2019), which required reporting and stewardship governance, could also have influenced the observed improvements (Supplementary Figure S10). However, ESBL-producing organisms increased sharply, from 30% of ICUs in 2017 to 50% in 2018, reflecting the global rise in ESBL endemicity.^20^^,^^25^ However, the consequent decrease in ESBL incidence probably reflects a shift in the AMR profile into CPE rather than a true decline in ESBL circulation. Regarding CPE emergence, while our findings show a low incidence throughout the study period when both adults and paediatric ICUs are analysed, there was a sharp increase in incidence in adults ICUs after the COVID-19 pandemic. Considering the stable CRE incidence, this shows a mechanistic change in carbapenem resistance, from a non-carbapenemase producing profile, explained mainly by ESBL production combined with porin alterations, to a carbapenemase-producing one. This data align with national reports from the Chilean Institute of Public Health showing CPE positivity rising sharply from 2% in 2014 to 16% in 2017,^26^ and across Latin America and the Caribbean, where 81% of 58,909 isolates tested between 2015 and 2020 carried a carbapenemase gene (mostly *bla*_KPC_ and *bla*_NDM_),^27^ with incidence surging after COVID-19 amid widespread empirical broad-spectrum ABU.^28^

ABU emerged as a substantial overall predictor of MDR, particularly for carbapenems and quinolones. These results mirror regional patterns: a multi-country study across 304 ICUs in nine Latin American nations reported antibiotic consumption of 15.3 DDD per 1000 patient-days, with carbapenems representing 22% of all prescriptions,^29^ highlighting their widespread use in critical care. In our study, overall carbapenem use was even higher (18.2 DDDs per 1000 bed-days), reinforcing their central role in resistance selection and incidence, a likely reason explaining these associations. Rising ESBL incidence increases carbapenem use as a therapeutic response, which in turn accelerates the emergence of carbapenemases (e.g., *bla*_KPC_, *bla*_NDM_). Furthermore, high quinolone use likely amplifies resistance spread through cross-resistance and plasmid-mediated gene dissemination, accounting for the observed patterns with greater MDR incidence. In fully adjusted drug-bug specific models, we found quinolone use positively associated with greater MRSA IDRs, consistent with evidence linking levofloxacin or ciprofloxacin exposure to selection of gene mutations and disruption of colonisation dynamics.^30^, ^31^, ^32^ Carbapenem use was significantly associated with higher incidence of CRE and CRPA, consistent with carbapenem exposure driving selection of strains with reduced outer membrane permeability, efflux pump overexpression, and β-lactamase derepression that collectively confer carbapenem-resistance in ICU settings.^33^

Institutional factors, including ICU type and hospital ownership, significantly influenced MDR IDRs. Paediatric ICUs and private/university hospitals were associated with lower MDR IDRs compared to adult ICUs and public hospitals, respectively. Private hospitals exhibited better adherence to infection control practices, infectious disease specialists and lower poverty-associated variables, likely contributing to their reduced AMR burden. These findings highlight the role of institutional characteristics in shaping AMR outcomes and suggest opportunities to transfer best practices across healthcare sectors. Paediatric ICUs had an overall incidence of 2.23 per 1000 patient-days, representing a 60.4% lower rate than that observed in adult populations. Private hospitals showed greater number of hours of infectious diseases specialists (e.g., an additional hour reduced MDR IDR by ∼2%) and lower incidence rates among most MDR combinations (e.g., CRAB, MRSA, ESBL), likely reflecting better hygiene compliance and infection control, since CRAB and MRSA are mainly driven by horizontal transmission, and rational antimicrobial use, given the role of selective pressure in ESBL emergence.^20^

Our study has some limitations. The ecological design precludes individual-level inference, and the reliance on aggregated data limits the ability to establish direct exposure–outcome relationships. Accordingly, findings should be interpreted with caution. However, following data from the Department of Health Statistics, our sample included the largest public hospitals, representing 65% of national public ICU capacity.^34^ Socioeconomic and demographic variables were derived from aggregated CASEN survey data, which may not fully capture heterogeneity at the institutional level or catchment area. Due to data limitations, we did not include other antibiotics relevant to ABU, such as vancomycin, which is important for VRE and MRSA. Moreover, critical data, such as invasive procedure rates (e.g., mechanical ventilation and catheter use) and the distinction between colonisation and infection, were unavailable, limiting the scope of the analysis. Also, for carbapenemase detection, participating hospitals adhered to national reference guidance and used validated phenotypic assays (e.g., Carba NP or Carba Blue); however, inter-hospital variability cannot be excluded. Finally, the study focused exclusively on ICUs, which, while high-risk environments, may not reflect MDR dynamics in other clinical settings, neither in the community. Compared to previous national analyses,^14^ we expanded the scope by including different drug-bug combinations, a larger number of hospitals, ABU data, and a higher resolution on hospital complexity (e.g., antimicrobial stewardship programs), detailed ICU-specific settings, and the most recent data available that were not yet explored. Interestingly, population-level poverty and migration were not significantly associated with MDR in our adjusted models. While this may reflect limitations in data granularity, it suggests that institutional factors, such as antibiotic stewardship and infection control practices, play a more direct role in determining MDR incidence than socioeconomic variables at the aggregated level (Supplementary Figure S10). However, these factors could not be directly assessed because the relevant data were not collected within the surveillance system.

Our findings identify key priorities to strengthen MDR control and inform Chile's National Action Plan on AMR. Expanding AMS coverage in public hospitals (i.e., half of the included public hospitals did not have one) and ensuring dedicated monitoring by infectious disease specialists are essential to strengthen stewardship and promote rational ABU. Reinforcing infection control and maintaining adherence to evidence-based protocols would further consolidate progress. These findings call for revising ICU ABU guidelines to kerb broad-spectrum and carbapenem overuse; practices shaped by high ESBL prevalence but now driving CRE spread, to better align with current epidemiological realities. This work advances UN SDG targets 3.8 and 3.d, fostering equitable access to quality antibiotics and resilient systems for MDR prevention and response.

## Contributors

Conceptualisation, M-PA, CC, KA, PR; methodology, M-PA, CC, KA; data collation and extraction, M-PA; formal analysis, M-PA, KA; writing—original draft preparation, M-PA, KA, PR; writing—review and editing, KA, M-PA, CC; supervision, CC, KA. All authors have read and approved the final version of the manuscript.

## Data sharing statement

IDR and ABU data are publicly available at https://sochinf.gcrb.cl/inicio/. Furthermore, the Supplemental Data file provides additional details on data sources.

## Editor disclaimer

The Lancet Group takes a neutral position with respect to territorial claims in published maps and institutional affiliations.

## Declaration of interests

The authors declare no conflict of interests.

## Appendix A Supplementary data

mSupplementary
