# Supplementary material for: Trends and drivers of multidrug-resistant bacteria incidence in 59 Chilean intensive care units, 2015–2024: a Bayesian hierarchical analysis
Source: Lancet Reg Health Am. 2026 Apr 4;58:101467. doi: 10.1016/j.lana.2026.101467 (PMC13089062; doi:10.1016/j.lana.2026.101467)
Supplement: mSupplementary [file mmc1.pdf]

Supplementary materials

Trends and Drivers of Multidrug-Resistant Bacteria Incidence in 59 Chilean Intensive Care Units, 2015–2024: A Bayesian Hierarchical Analysis

Table of contents

Glossary of abbreviations 3

Table S1. Name of the participating centres 4

Table S2. Municipalities belonging to each Health Service included in the study (Source: DEIS) 5

Text S1. Inclusion / exclusion criteria for microbiological isolates 6

Table S3. Number of isolates per antibiotic-resistant/pathogen specific combination in the included ICU settings, over time 7

Table S4: Response Variable – Incidence Density Rate per 1,000 Patient-Days for 8 Bacteria of Critical or High Priority According to WHO Pathogen priority list 8

Table S5: Explanatory Variables Related to Healthcare Centres and ICU Services 9

Table S6: Explanatory Variables Related to Antibiotic Consumption 10

Table S7: Socioeconomic and Demographic Explanatory Variables of the Population Served by Healthcare Institutions 11

Table S8: Bayesian hierarchical Diagram and Number of Observations at Each Level 12

Figure S1. Structure of our study data separated by adult and paediatric ICUs 13

Figure S2. Directed Acyclic Graph 14

Table S9. Number of participating ICUs over time period 14

Figure S3. Temporal incidence trends of major antimicrobial-resistant pathogens in Chilean ICUs among adults, 2015-2024 15

Figure S4. Temporal incidence trends of major antimicrobial-resistant pathogens in Chilean ICUs among paediatrics, 2015-2024 16

Figure S5. Average incidence density rate per 1,000 patient-days of major antimicrobial-resistant pathogens in Chilean ICUs among adults, 2015-2024 17

Figure S6. Average incidence density rate per 1,000 patient-days of major antimicrobial-resistant pathogens in Chilean ICUs among paediatrics, 2015-2024 18

Figure S7. Temporal incidence trends of major antimicrobial-resistant pathogens in public Chilean ICUs, 2015-2024 19

Figure S8. Temporal incidence trends of major antimicrobial-resistant pathogens in private Chilean ICUs, 2015-2024 20

Figure S9. Posterior estimates from a Bayesian hierarchical model evaluating the impact of antibiotic use on the incidence of multidrug-resistant infections in ICU settings, 2015–2024, after adjustment for confounders 21

Table S10. Estimated time- and antibiotic-use-adjusted incidence from Bayesian hierarchical models of multidrug-resistant infections in ICU settings (2015-2024), stratified by drug-bug combinations 22

Table S11. Results from Bayesian hierarchical models assessing antibiotic use as the main determinant of multidrug-resistant infection incidence in ICU settings, 2015-2024, by bug-drug combination 24

Figure S10. Key AMR and policy IPC milestones in Chile over recent years 25

STROBE Guidelines 26

Glossary of abbreviations

AMR= Antimicrobial resistance
ABU= Antibiotic use
CASEN= *Encuesta de Caracterización Socioeconómica Nacional* or the National Socioeconomic Characterization Survey.
CI= Confidence interval
CPE= Carbapenemase producing Enterobacterales
CRAB= Carbapenem-resistant *Acinetobacter baumannii*
CRE= Carbapenem-resistant Enterobacterales
CRPA= Carbapenem-resistant *Pseudomonas aeruginosa*
DDD= Daily defined doses
DEIS= Department of Health Statistics
ESBL= Extended-spectrum beta-lactamase producing
GCRB= Collaborative Group on Bacterial Resistance or Grupo Colaborativo de Resistencia Bacteriana.
ICC= Intraclass correlation
ICU= Intensive care units
IQR= Interquartile range
MRSA= Methicillin-resistant *Staphylococcus aureus*
MDR= Multidrug resistance
PAHO= Panamerican Health Organization
VRE= Vancomycin-resistant Enterococcus species
WHO= World Health Organization

Table S1. Name of the participating centres

1. H. de Iquique Dr. Ernesto Torres Galdames	2. H. Clínico Red de Salud UC Christus
3. H. Regional Dr. Leonardo Guzmán de Antofagasta	4. Complejo Hospitalario San José
5. H. Dr. Eduardo Pereira Ramírez	6. H. Roberto del Río
7. H. Dr. Gustavo Fricke	8. H. San Juan de Dios
9. H. Dr. Franco Ravera Zunino de Rancagua	10. H. Clínico San Borja Arriarán
11. H. Regional de Talca	12. H. del Salvador
13. H. Clínico Regional de Concepción, Dr. Guillermo Grant Benavente	14. Instituto Nacional del Tórax
15. H. Las Higueras	16. H. Luis Calvo Mackenna
17. H. Clínico Herminda Martin, Chillán	18. Complejo Asistencial Barros Luco Trudeau
19. Complejo Asistencial Dr. Víctor Ríos Ruiz, Los Ángeles	20. H. de Enfermedades Infecciosas Dr. Lucio Córdova
21. H. Dr. Hernán Henríquez Aravena, Temuco	22. H. Exequiel González Cortés
23. H. Base de Valdivia	24. Complejo Asistencial Dr. Sótero del Río
25. H. Base San José de Osorno	26. H. Padre Hurtado
27. H. de Puerto Montt Dr. Eduardo Schütz Schroeder	28. H. Clínico Dra. Eloísa Díaz I. La Florida
29. H. Clínico Magallanes Dr. Lautaro Navarro Avaria	30. Bupa Clínica Reñaca
31. H. de la Dirección de Previsión de Carabineros de Chile (DIPRECA)	32. Clínica Alemana de Santiago
33. H. Clínico de la Fuerza Aérea de Chile (FACH)	34. Clínica Las Condes
35. H. Militar de Santiago	36. Clínica Dávila
37. H. Naval Almirante Nef	38. Clínica RedSalud Santiago
39. H. Clínico Universidad de Chile	40. Clínica San Carlos de Apoquindo UC Christus

Notes: H.= Hospital

Table S2. Municipalities belonging to each Health Service included in the study (Source: DEIS)

Health service	Municipality
Iquique y Tarapacá	Iquique, Camiña, Colchane, Huara, Pica, Pozo Almonte, Alto Hospicio
Antofagasta	Antofagasta, Mejillones, Sierra Gorda, Taltal, Calama, San Pedro de Atacama, Ollagüe, Tocopilla, María Elena
Valparaíso – San Antonio	Valparaíso, Casablanca, Juan Fernández, San Antonio, Algarrobo, Cartagena, Santo Domingo, El Quisco, El Tabo.
Viña del Mar – Quillota	Concón, Puchuncaví, Quilpué, Quintero, Villa Alemana, Viña del Mar, La Ligua, Cabildo, Papudo, Petorca, Zapallar, Quillota, Calera, Hijuelas, La Cruz, Limache, Nogales, Olmué
Metropolitano Norte	Conchalí, Huechuraba, Independencia, Quilicura, Recoleta, Colina, Lampa, Tiltil
Metropolitano Occidente	Cerro Navia, Lo Prado, Pudahuel, Quinta Normal, Renca, Melipilla, Alhué, Curacaví, María Pinto, San Pedro, Talagante, El Monte, Isla Maipo, Padre Hurtado, Peñaflor
Metropolitano Central	Santiago, Cerrillos, Estación Central, Maipú
Metropolitano Oriente	Isla de Pascua, La Reina, Las Condes, Lo Barnechea, Macul, Ñuñoa, Peñalolén, Providencia, Vitacura
Metropolitano Sur	El Bosque, La Cisterna, Lo Espejo, Pedro Aguirre Cerda, San Joaquín, San Miguel, San Bernardo, Buin, Calera de Tango, Paine
Metropolitano Sur Oriente	La Florida, La Granja, San Ramón, La Pintana, Puente Alto, San José de Maipo, Pirque
O'Higgins	Rancagua, Codegua, Coinco, Coltauco, Doñihue, Graneros, Las Cabras, Machalí, Malloa, Mostazal, Olivar, Peumo, Pichidegua, Quinta de Tilcoco, Rengo, Requínoa, San Vicente, Pichilemu, La Estrella, Litueche, Marchihue, Navidad, Paredones, San Fernando, Chépica, Chimbarongo, Lolol, Nancagua, Palmilla, Peralillo, Placilla, Pumanque, Santa Cruz
Del Maule	Talca, Constitución, Curepto, Empedrado, Maule, Pelarco, Pencahue, Río Claro, San Clemente, San Rafael, Cauquenes, Chanco, Pelluhue, Curicó, Hualañé, Licantén, Molina, Rauco, Romeral, Sagrada Familia, Teno, Vichuquén, Linares, Colbún, Longaví, Parral, Retiro, San Javier, Villa Alegre, Yerbas Buenas
Concepción	Concepción, Coronel, Chiguayante, Florida, Hualqui, Lota, San Pedro de la Paz, Santa Juana
Talcahuano	Talcahuano, Tomé, Hualpén, Penco
Bío Bío	Los Ángeles, Antuco, Cabrero, Laja, Mulchén, Nacimiento, Negrete, Quilaco, Quilleco, San Rosendo, Santa Bárbara, Tucapel, Yumbel, Alto Biobío
Araucanía Sur	Temuco, Carahue, Cunco, Curarrehue, Freire, Galvarino, Gorbea, Lautaro, Loncoche, Melipeuco, Nueva Imperial, Padre Las Casas, Perquenco, Pitrufquén, Pucón, Saavedra, Teodoro Schmidt, Toltén, Vilcún, Villarrica, Cholchol
Valdivia	Valdivia, Corral, Futrono, La Unión, Lago Ranco, Lanco, Los Lagos, Máfil, Mariquina, Paillaco, Panguipulli, Río Bueno
Del Reloncaví	Puerto Montt, Calbuco, Cochamó, Fresia, Frutillar, Los Muermos, Llanquihue, Maullín, Puerto Varas, Chaitén, Futaleufú, Hualalhue, Palena
Magallanes	Punta Arenas, Laguna Blanca, Río Verde, San Gregorio, Cabo de Hornos, Antártica, Porvenir, Primavera, Timaukel, Natales, Torres del Paine

Text S1. Inclusion / exclusion criteria for microbiological isolates

Inclusion criteria

- Isolates from patients hospitalized in adult ICUs (surgical, medical, medical-surgical, or general) and pediatric ICUs. A hospital may report data for both adult and pediatric ICUs, but the information must be submitted separately.
- Only one isolate per patient for each bacterium with the specified resistance pattern.
- The isolate must be the first isolate of each bacterium recorded during the study period.
- If a patient has more than one positive culture for the same bacterium within six months, only the first will be considered.
- A single patient may have more than one species of multidrug-resistant bacteria.
- Cultures obtained from clinical samples representing either infection or colonization will be considered.
- Microbiological criteria for isolates, according to CLSI definitions (using 2018 as exemplar but we adapt them on a year basis), as follows:
  - **MRSA**: cefoxitin 30 µg disc diffusion zone ≤ 21 mm or cefoxitin MIC ≥ 8.
  - **VRE**: vancomycin 30 µg disc diffusion zone ≤ 14 mm or vancomycin MIC ≥ 32.
  - **ESBL**: combination of ceftazidime 30 µg, cefotaxime 30 µg, ceftazidime/clavulanate 30/10 µg, cefotaxime/clavulanate 30/10 µg, with an increase in inhibition zone ≥ 5 mm. Or by MIC, defined as an increase of ≥ 3 dilutions in MIC for each antibiotic/clavulanate combination compared with the antibiotic alone.
  - **Carbapenem-resistant Enterobacteriaceae**: ertapenem zone ≤ 18 mm or imipenem ≤ 19 mm or meropenem ≤ 19 mm. Or by MIC: ertapenem ≥ 2, imipenem ≥ 4, meropenem ≥ 4. Any Enterobacteriaceae resistant to at least one carbapenem is considered, regardless of the probable resistance mechanism.
  - **Carbapenem-resistant P. aeruginosa**: imipenem 10 µg disc zone ≤ 15 mm or meropenem 10 µg ≤ 15 mm. Or by MIC: imipenem ≥ 8, meropenem ≥ 8.
  - **A. baumannii**: imipenem 10 µg disc zone ≤ 18 mm or meropenem 10 µg ≤ 14 mm. Or by MIC: imipenem ≥ 8, meropenem ≥ 8.
  - **Carbapenemase-producing Enterobacterales**: Carbapenemase-producing Enterobacterales confirmed using phenotypic or molecular testing methods.

Exclusion criteria

- Intermediate care units, neonatal units, coronary care units, or burn ICUs are excluded.
- Results of cultures obtained for epidemiological surveillance purposes are excluded.

Table S3. Number of isolates per antibiotic-resistant/pathogen specific combination in the included ICU settings, over time

	N isolates							
**Year**	**MRSA**	**VRE**	**ESCO**	**KLPN**	**CRE**	**CPE**	**CRPA**	**CRAB**
2015	403	157	190	431	204	NA	501	226
2016	774	202	364	854	508	NA	734	371
2017	724	282	374	879	540	25	779	236
2018	583	287	415	1,015	657	95	805	208
2021	591	485	351	1,412	856	188	670	214
2023	360	536	530	986	1,012	468	636	55
2024	503	668	636	891	942	377	690	42
TOTAL	3,938	2,617	2,860	6,468	4,719	1,153	4,815	1,352

Notes: MRSA: methicillin-resistant Staphylococcus aureus; VRE: vancomycin-resistant Enterococcus; ESCO: extended-spectrum β-lactamase (ESBL)-producing Escherichia coli; KLPN: extended-spectrum β-lactamase (ESBL)-producing Klebsiella pneumoniae; CRE: carbapenem-resistant Enterobacterales; CPE: carbapenemase-producing Enterobacterales; CRPA: carbapenem- resistant Pseudomonas aeruginosa; CRAB: carbapenem- resistant Acinetobacter baumannii. *NA: not available. Data collection began in 2017

Table S4: Response Variable – Incidence Density Rate per 1,000 Patient-Days for 8 Bacteria of Critical or High Priority According to WHO Pathogen priority list

Name	Definition	Operationalization
**MRSA**	Incidence of methicillin-resistant *Staphylococcus aureus* (MRSA) per 1,000 patient-days in each reported ICU	(Number of MRSA isolates in ICU ÷ ICU bed-days occupied) × 1,000
**VRE**	Incidence of vancomycin-resistant *Enterococcus* (VRE) per 1,000 patient-days in each reported ICU	(Number of VRE isolates in ICU ÷ ICU bed-days occupied) × 1,000
**ESBL KP**	Incidence of *Klebsiella pneumoniae* ESBL per 1,000 patient-days in each reported ICU	(Number of ESBL KP isolates in ICU ÷ ICU bed-days occupied) × 1,000
**ESBL EC**	Incidence of *Escherichia coli* ESBL per 1,000 patient-days in each reported ICU	(Number of ESBL EC isolates in ICU ÷ ICU bed-days occupied) × 1,000
**CRE**	Incidence of carbapenem-resistant Enterobacteriaceae (CRE) per 1,000 patient-days in each reported ICU	(Number of CRE isolates in ICU ÷ ICU bed-days occupied) × 1,000
**CPE**	Incidence of carbapenemase-producing Enterobacteriaceae (CPE) per 1,000 patient-days in each reported ICU	(Number of CPE isolates in ICU ÷ ICU bed-days occupied) × 1,000
**CRPA**	Incidence of carbapenem-resistant *Pseudomonas aeruginosa*(CRPA) per 1,000 patient-days in each reported ICU	(Number of CRPA isolates in ICU ÷ ICU bed-days occupied) × 1,000
**CRAB**	Incidence of carbapenem-resistant *Acinetobacter baumannii*(CRAB) per 1,000 patient-days in each reported ICU	(Number of CRAB isolates in ICU ÷ ICU bed-days occupied) × 1,000

Notes: MRSA: Methicillin-resistant *Staphylococcus aureus*.VRE: Vancomycin-resistant Enterococcus. 3GCRKP: *Klebsiella pneumoniae* resistant to third-generation cephalosporins. 3GCRESCO: Escherichia coli resistant to third-generation cephalosporins. CRE: Carbapenem-resistant Enterobacteriaceae. CPE: Carbapenemase-producing Enterobacteriaceae. CRPA: Carbapenem-resistant *Pseudomonas aeruginosa*. CRAB: Carbapenem-resistant *Acinetobacter baumannii*.

Table S5: Explanatory Variables Related to Healthcare Centres and ICU Services

Name	Definition	Operationalization
**Hospital type (type_hosp)**	Classification of the institution by administration type	1 = Public; 2 = Private or University; 3 = Institutional (e.g., Armed Forces and Order)
**Total hospital beds (beds_total)**	Total number of beds in the institution during the study year	-
**ICU beds (beds_ICU)**	Number of ICU beds in the institution during the study year	-
**Cardiac surgery (cardioqx)**	Centers providing cardiac surgery services as defined by the FONASA Services Catalog	0 = No; 1 = Yes
**Neurosurgery (neuroqx)**	Centers providing neurosurgery services as defined by the FONASA Services Catalog	0 = No; 1 = Yes
**Hematology-oncology (hemato_qmt)**	Centers treating patients with hematological neoplasms	0 = No; 1 = Yes
**Infectious disease hours (infecto_bed)**	Contracted hours of infectious disease specialists per 100 beds	(Number of contracted ID specialist hours × 100) ÷ Total hospital beds
**Antimicrobial stewardship program (PROA)**	Implementation of an antimicrobial stewardship program	0 = No; 1 = Yes

Notes: ICU= Intensive care units.

Table S6: Explanatory Variables Related to Antibiotic Consumption

Name	Definition	Calculation
**Cephalosporin DDD (DDD_cef)**	Consumption of third- and fourth-generation cephalosporins (e.g., cefotaxime, ceftriaxone) in DDD	(Total DDD of cephalosporins in ICU ÷ Occupied ICU bed-days) × 100
**Quinolone DDD (DDD_quin)**	Consumption of quinolones (e.g., ciprofloxacin, levofloxacin) in DDD	(Total DDD of quinolones in ICU ÷ Occupied ICU bed-days) × 100
**Carbapenem DDD (DDD_carba)**	Consumption of carbapenems (e.g., ertapenem, meropenem) in DDD	(Total DDD of carbapenems in ICU ÷ Occupied ICU bed-days) × 100

Notes: DDD= Daily defined dosis. ICU= Intensive care units.

Table S7: Socioeconomic and Demographic Explanatory Variables of the Population Served by Healthcare Institutions

**Name**	**Definition**
**Older adults (older_adults)**	Percentage of the population assigned to the institution aged 60 years or older.
**Rural population (rural)**	Percentage of the population assigned to the institution living in rural areas.
**Poverty (poverty)**	Percentage of the population classified as living in poverty based on household income.
**Extreme poverty (extreme_poverty)**	Percentage of the population classified as living in extreme poverty based on household income.
**Multidimensional poverty (4 dimensions) (multi_poverty4)**	Percentage of the population in multidimensional poverty (education, health, work, housing).
**Multidimensional poverty (5 dimensions) (multi_poverty5)**	Percentage of the population in multidimensional poverty (education, health, work, housing, social cohesion).
**Overcrowding (overcrowding)**	Percentage of the population living in overcrowded conditions.
**Severe overcrowding (severe_overcrowding)**	Percentage of the population living in critically overcrowded conditions.
**Migrants (migrants)**	Percentage of the population with non-Chilean nationality assigned to the institution.
**Recent migration (recent_migration)**	Percentage of the population with recent international migration (based on residence 5 years prior).
**Access to basic services (sanitation)**	Percentage of the population with limited access to basic services (e.g., water, hygiene, sanitation).

Notes:

Table S8: Bayesian hierarchical Diagram and Number of Observations at Each Level

Level	Level Index	Identification Variable	Variables Measured at Each Level
**Centers (i = 40)**	i = 1,…, 40	hospital_id	Hospital type (type_hospitali), neurosurgery (neurocxi), cardiac surgery (cardiocxi), hematology (hematoi), antimicrobial stewardship program (PROAi)total beds (beds_totalibeds_totali), infectious disease specialist hours (infectology_hoursi)
**Bacteria (j = 8)**	j = 1,…, 8	bacteria	-
**Time (t = 4)**	t = 1,…, 4	time	Incidence density rate (IDT) for: MRSA (IDR_MRSAij), VRE (IDR _ERVijt), 3GCR E. coli (IDR _ESCOijt), 3GCR K. pneumoniae (IDR _KLPNijt), CRE (IDR _ERCijt), CRPA (TDI_PSAEijtTDI_PSAEijt), CRAB (IDR _ACBAijtTDI_ACBAijt), CPE (IDR _EPCijtTDI_EPCijt); antibiotic consumption (DDDijt); invasive procedures: VMI days (VMIijt), urinary catheter days (CUPijt), central venous catheter days (CVCijt) sociodemographics: rurality (ruralityijt), age (ageijt), migration (migrationijt), poverty (povertyijt), overcrowding (overcrowdingijt), sanitation (sanitationijt).

Figure S1. Structure of our study data separated by adult and paediatric ICUs

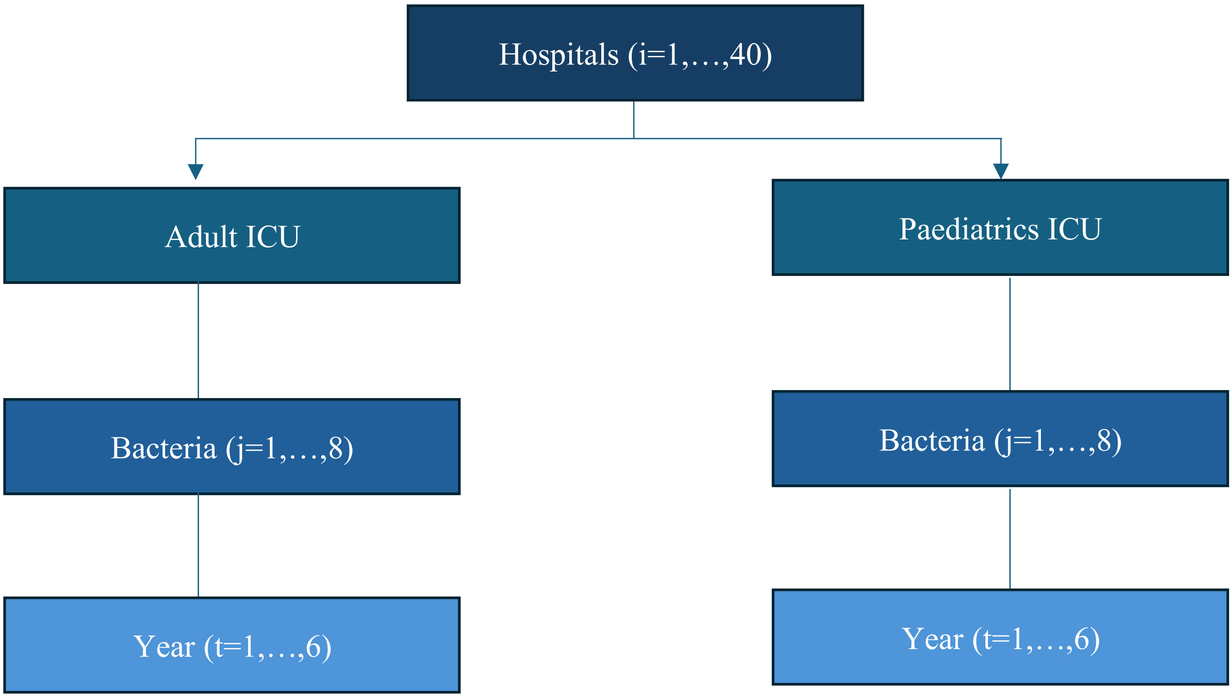

Notes: ICU= Intensive care unit. Values are taken as examples.

Figure S2. Directed Acyclic Graph

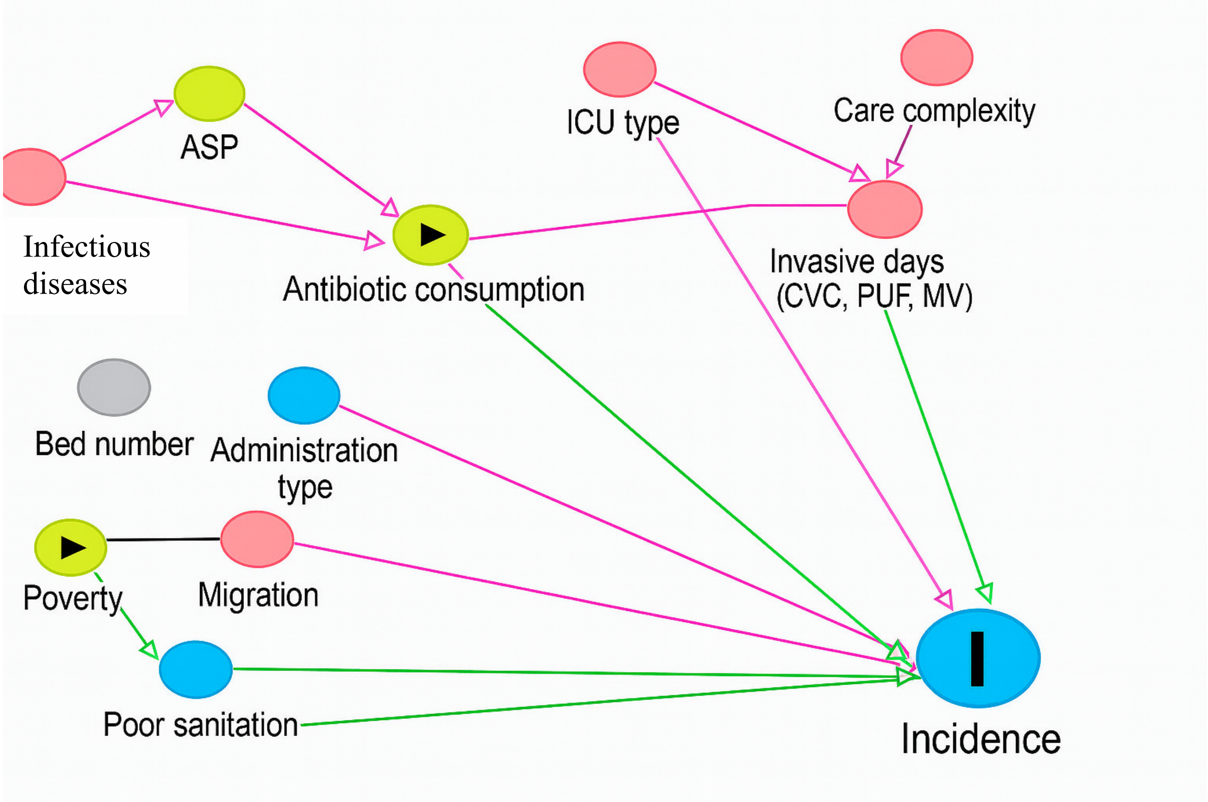

Notes: ICU= Intensive care unit, MV= mechanical ventilation. ASP= Antimicrobial stewardship.

Table S9. Number of participating ICUs over time period

Year	Number of ICUs
2015	28
2016	34
2017	38
2018	49
2021	23
2023	33
2024	38

Notes: ICU= Intensive care units.

Figure S3. Temporal incidence trends of major antimicrobial-resistant pathogens in Chilean ICUs among adults, 2015-2024

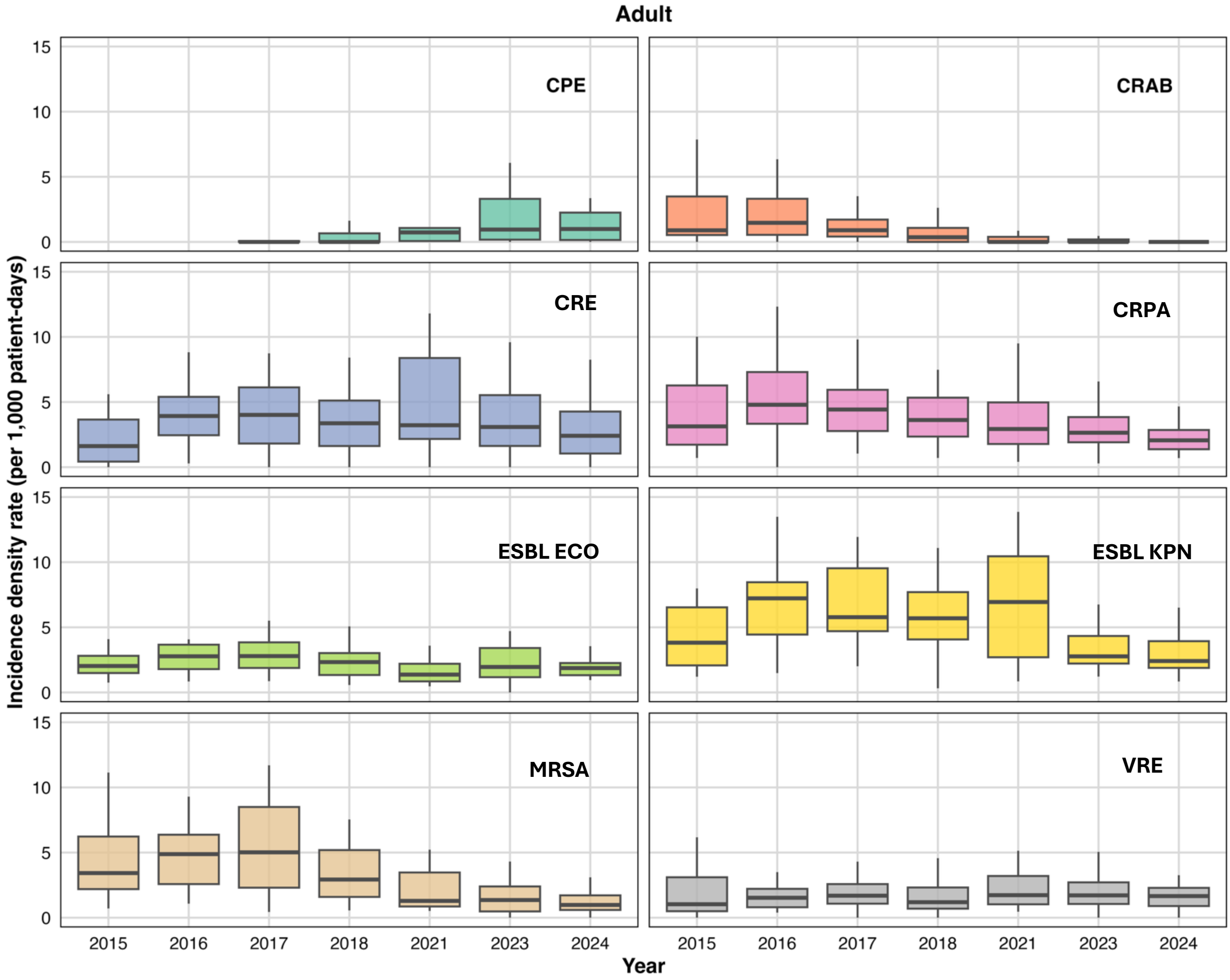

Notes: ICU= Intensive care unit. We removed 2019 and 2020 because of misreporting due to the COVID-19 pandemic. CPE= Carbapenemase-producing Enterobacterales. CRAB=Carbapenem-resistant *Acinetobacter baumannii*. CREC= Carbapeem-resistant Enterobacterales. CRPA= Carbapenem-resistant *Pseudomonas aeruginosa*. ESBL ECO= Extended spectrum beta-lactamase producing *Escherichia coli*. ESBL ECO= Extended spectrum beta-lactamase producing *Klebsiella pneumoniae.* MRSA= Methicillin-resistant *Staphylococcus aureus.* VRE= Vancomycin-resistant Enterococcus species.

Figure S4. Temporal incidence trends of major antimicrobial-resistant pathogens in Chilean ICUs among paediatrics, 2015-2024

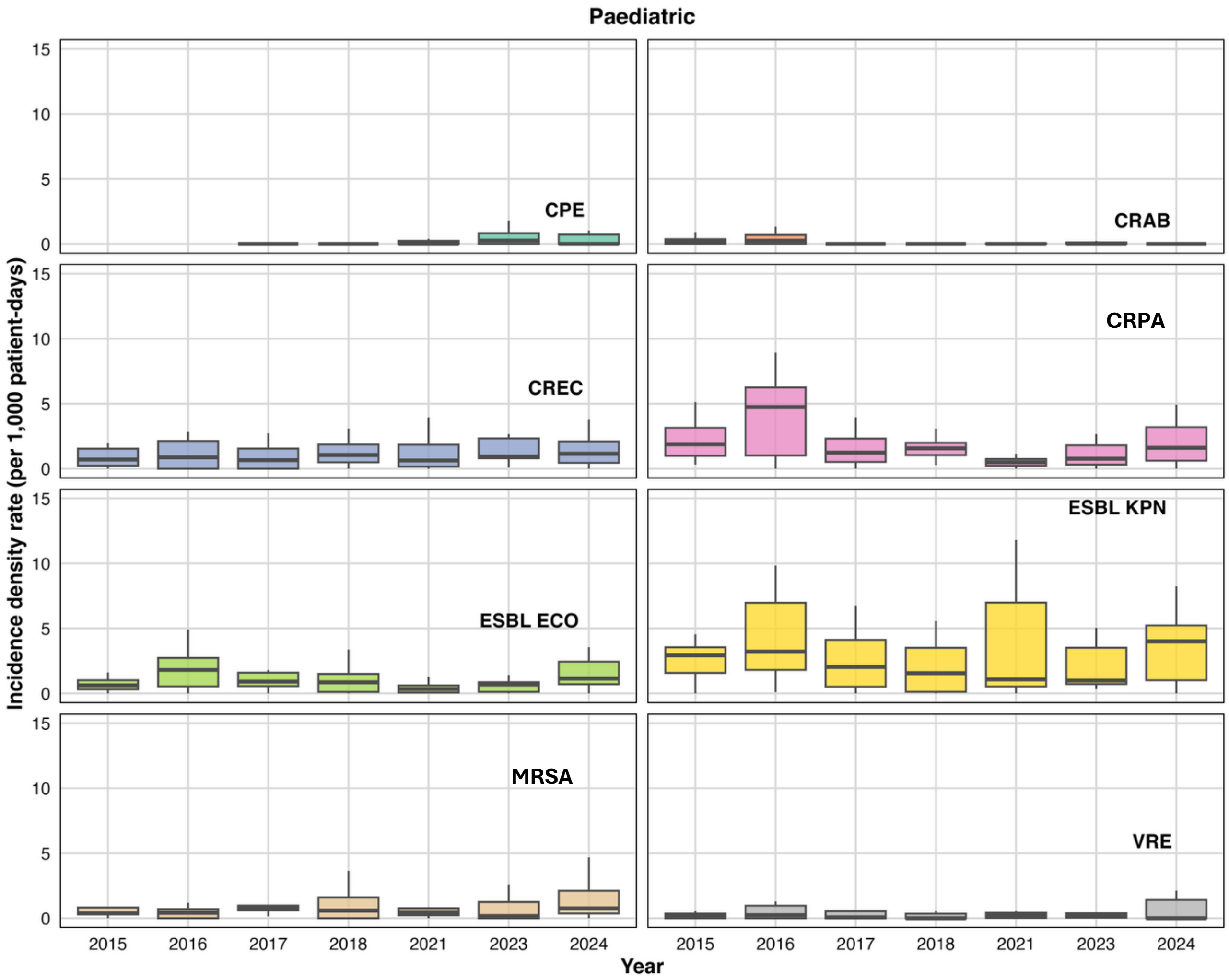

Notes: ICU= Intensive care unit. We removed 2019 and 2020 because of misreporting due to the COVID-19 pandemic. CPE= Carbapenemase-producing Enterobacterales. CRAB=Carbapenem-resistant *Acinetobacter baumannii*. CREC= Carbapeem-resistant Enterobacterales. CRPA= Carbapenem-resistant *Pseudomonas aeruginosa*. ESBL ECO= Extended spectrum beta-lactamase producing *Escherichia coli*. ESBL ECO= Extended spectrum beta-lactamase producing *Klebsiella pneumoniae.* MRSA= Methicillin-resistant *Staphylococcus aureus.* VRE= Vancomycin-resistant Enterococcus species.

Figure S5. Average incidence density rate per 1,000 patient-days of major antimicrobial-resistant pathogens in Chilean ICUs among adults, 2015-2024

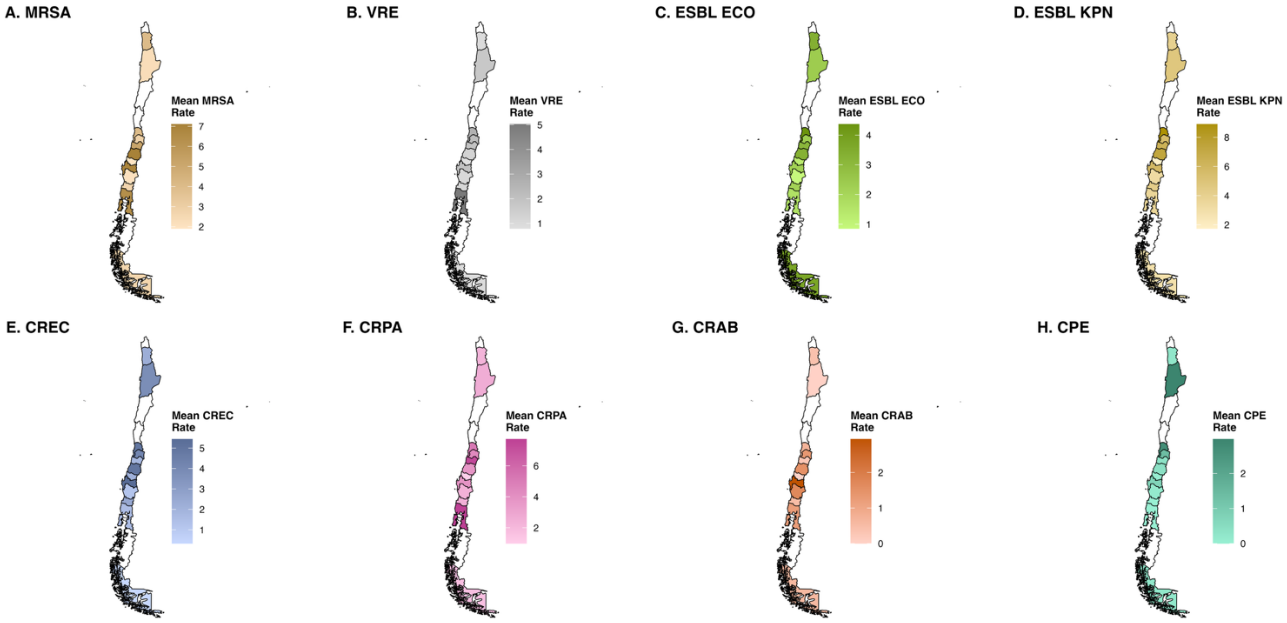

Notes: The average resistance rate is presented in maps using the mean rate of the incidence density rate per 1,000 patient-days. White areas (regions) indicate no hospital data available. ICU= Intensive care unit. We removed 2019 and 2020 because of misreporting due to the COVID-19 pandemic. CPE= Carbapenemase-producing Enterobacterales. CRAB=Carbapenem-resistant *Acinetobacter baumannii*. CREC= Carbapeem-resistant Enterobacterales. CRPA= Carbapenem-resistant *Pseudomonas aeruginosa*. ESBL ECO= Extended spectrum beta-lactamase producing *Escherichia coli*. ESBL KPN= Extended spectrum beta-lactamase producing *Klebsiella pneumoniae.* MRSA= Methicillin-resistant *Staphylococcus aureus.* VRE= Vancomycin-resistant *Enterococcus* species.

Figure S6. Average incidence density rate per 1,000 patient-days of major antimicrobial-resistant pathogens in Chilean ICUs among paediatrics, 2015-2024

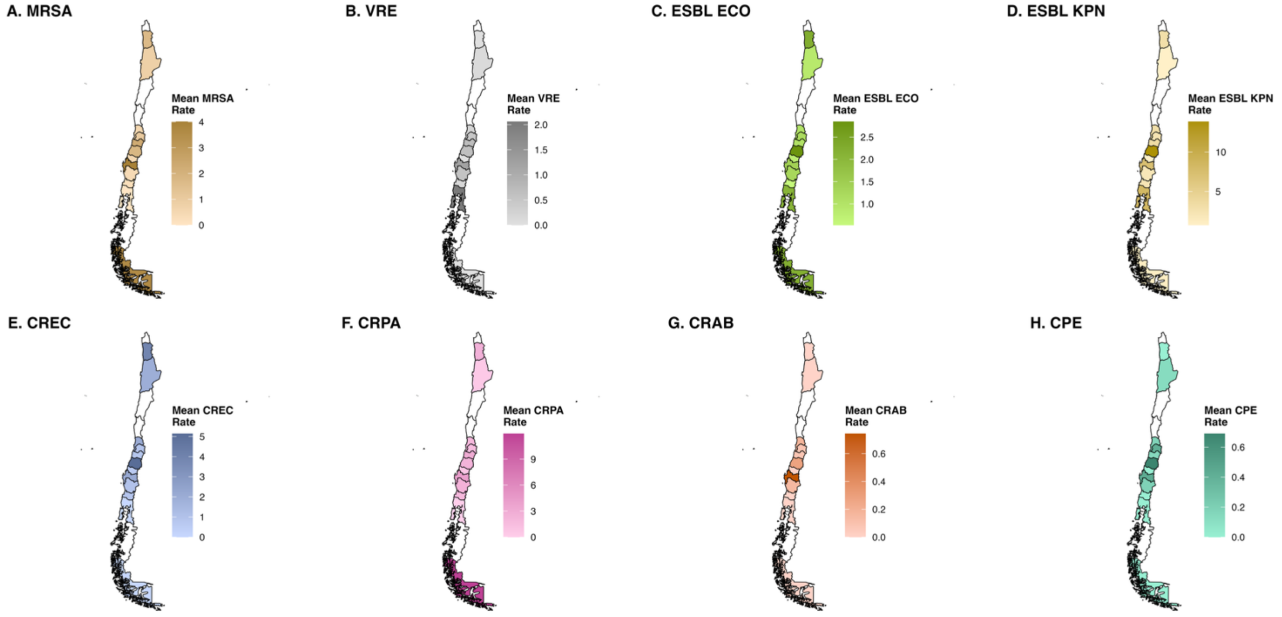

Notes: The average resistance rate is presented in maps using the mean rate of the incidence density rate per 1,000 patient-days. White areas (regions) indicate no hospital data available. ICU= Intensive care unit. We removed 2019 and 2020 because of misreporting due to the COVID-19 pandemic. CPE= Carbapenemase-producing Enterobacterales. CRAB=Carbapenem-resistant *Acinetobacter baumannii*. CREC= Carbapeem-resistant Enterobacterales. CRPA= Carbapenem-resistant *Pseudomonas aeruginosa*. ESBL ECO= Extended spectrum beta-lactamase producing *Escherichia coli*. ESBL KPN= Extended spectrum beta-lactamase producing *Klebsiella pneumoniae.* MRSA= Methicillin-resistant *Staphylococcus aureus.* VRE= Vancomycin-resistant *Enterococcus* species.

Figure S7. Temporal incidence trends of major antimicrobial-resistant pathogens in public Chilean ICUs, 2015-2024

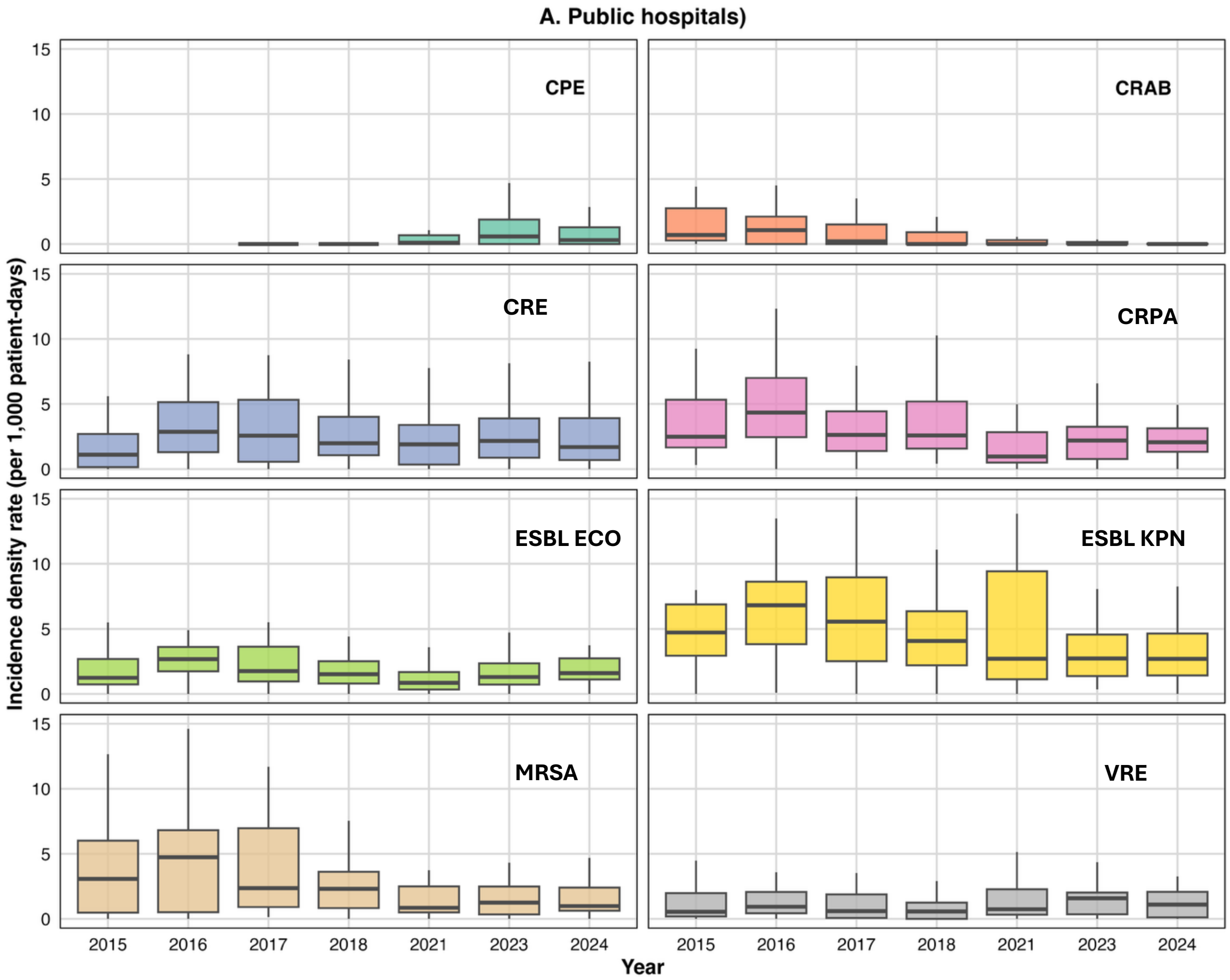

Notes: ICU= Intensive care unit. We removed 2019 and 2020 because of misreporting due to the COVID-19 pandemic. CPE= Carbapenemase-producing Enterobacterales. CRAB=Carbapenem-resistant *Acinetobacter baumannii*. CREC= Carbapeem-resistant Enterobacterales. CRPA= Carbapenem-resistant *Pseudomonas aeruginosa*. ESBL ECO= Extended spectrum beta-lactamase producing *Escherichia coli*. ESBL ECO= Extended spectrum beta-lactamase producing *Klebsiella pneumoniae.* MRSA= Methicillin-resistant *Staphylococcus aureus.* VRE= Vancomycin-resistant Enterococcus species.

Figure S8. Temporal incidence trends of major antimicrobial-resistant pathogens in private Chilean ICUs, 2015-2024

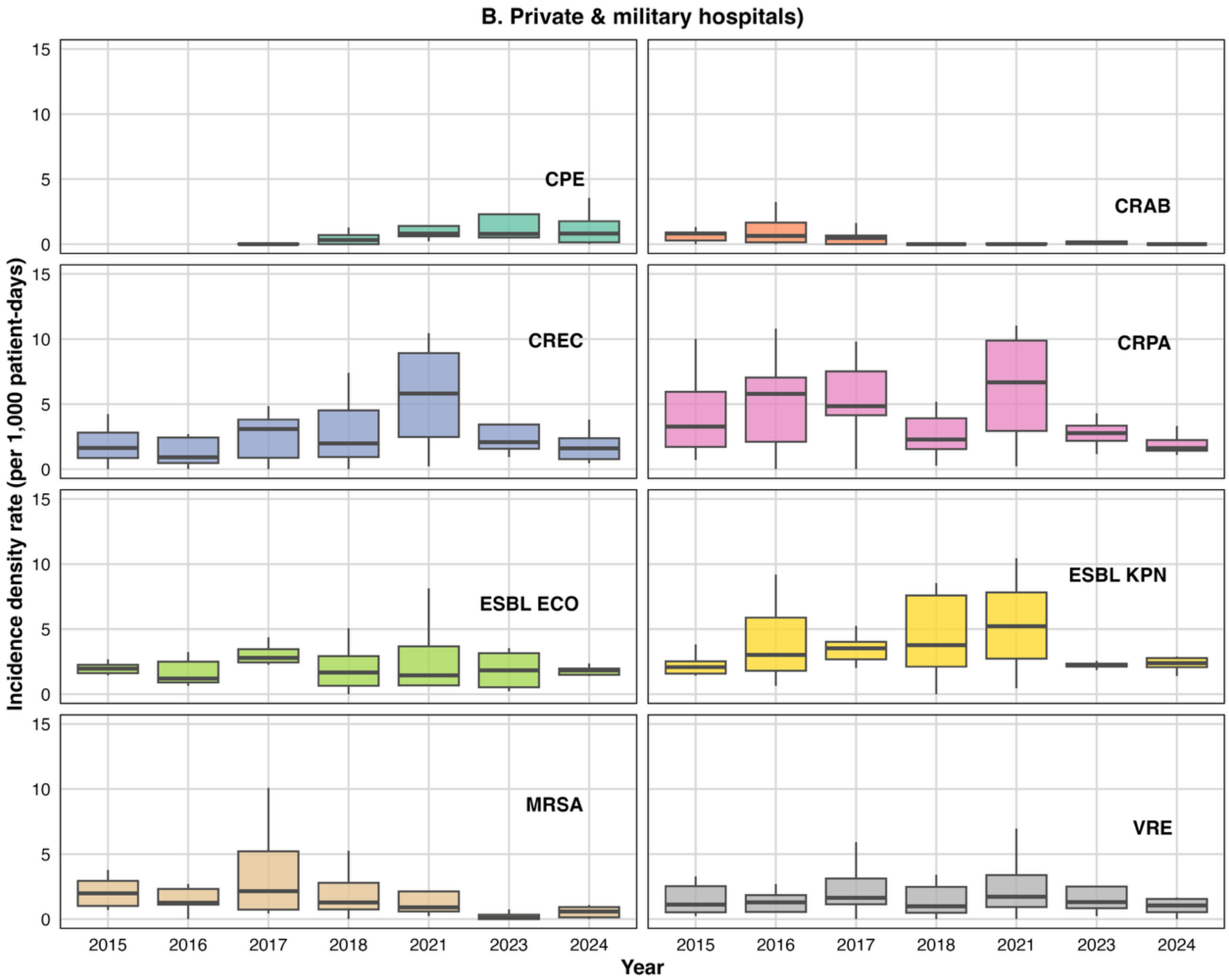

Notes: ICU= Intensive care unit. We removed 2019 and 2020 because of misreporting due to the COVID-19 pandemic. CPE= Carbapenemase-producing Enterobacterales. CRAB=Carbapenem-resistant *Acinetobacter baumannii*. CREC= Carbapeem-resistant Enterobacterales. CRPA= Carbapenem-resistant *Pseudomonas aeruginosa*. ESBL ECO= Extended spectrum beta-lactamase producing *Escherichia coli*. ESBL ECO= Extended spectrum beta-lactamase producing *Klebsiella pneumoniae.* MRSA= Methicillin-resistant *Staphylococcus aureus.* VRE= Vancomycin-resistant Enterococcus species.

Figure S9. Posterior estimates from a Bayesian hierarchical model evaluating the impact of antibiotic use on the incidence of multidrug-resistant infections in ICU settings, 2015–2024, after adjustment for confounders

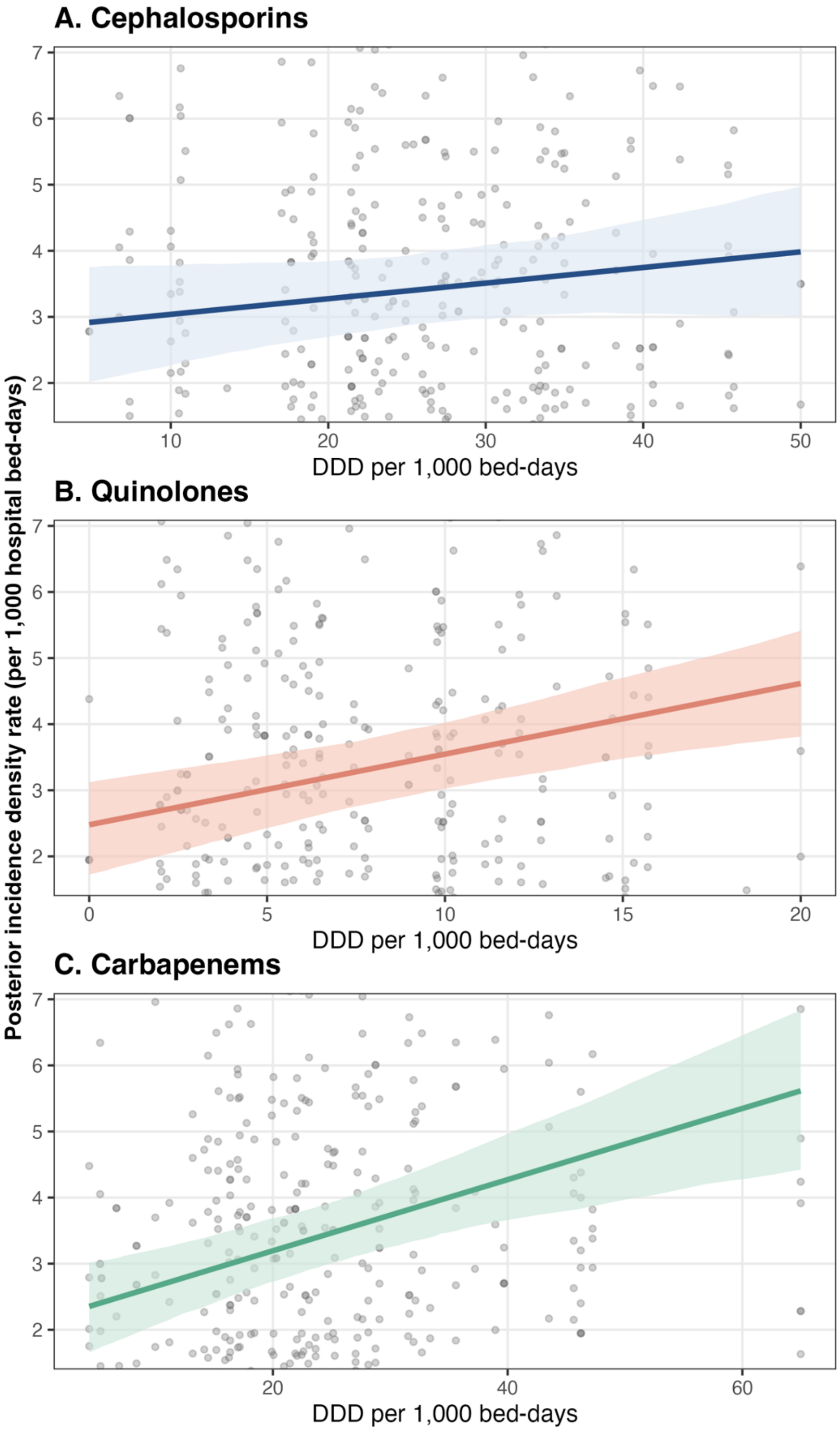

Notes: No collinearity problem, correlation was very low (Pearson *r* <0.3). Predicted incidence density rate was calculated among all pathogen-antibiotic combinations.

Table S10. Estimated time- and antibiotic-use-adjusted incidence from Bayesian hierarchical models of multidrug-resistant infections in ICU settings (2015-2024), stratified by drug-bug combinations

Variable	**MRSA** Coeff. [95%CI] [*p*]	**VRE** Coeff. [95%CI] [*p*]	**ESBL EC** Coeff. [95%CI] [*p*]	**ESBL KPN** Coeff. [95%CI] [*p*]	**CRE** Coeff. [95%CI] [*p*]	**CRPA** Coeff. [95%CI] [*p*]	**CRAB** Coeff. [95%CI] [*p*]	**CPE** Coeff. [95%CI] [*p*]
**A. Cephalosporins use**								
Intercept	4.54 (2.59–6.45) [<0.001]	2.33 (0.91–3.74) [0.003]	2.57 (1.07–4.07) [0.001]	5.57 (3.00–8.12) [<0.001]	2.13 (-0.22–4.45) [0.075]	3.85 (1.64–6.07) [<0.001]	1.55 (0.52–2.57) [0.004]	-0.47 (-2.29–1.31) [0.601]
Time [year]	-0.80 (-1.05–-0.55) [0.000]	0.08 (-0.10–0.26) [0.380]	0.02 (-0.17–0.21) [0.851]	-0.28 (-0.59–0.05) [0.096]	0.13 (-0.17–0.45) [0.382]	-0.31 (-0.59–-0.03) [0.031]	-0.42 (-0.55–-0.29) [<0.001]	0.43 (0.16–0.69) [0.002]
Cephalosporins use in DDD per 1,000 hospital bed-days	0.06 (0.00–0.12) [0.037]	-0.02 (-0.06–0.02) [0.297]	-0.00 (-0.05–0.04) [0.991]	0.02 (-0.06–0.10) [0.596]	0.04 (-0.03–0.11) [0.291]	0.04 (-0.02–0.11) [0.185]	0.03 (0.00–0.06) [0.036]	-0.01 (-0.06–0.04) [0.749]
σ^2	7.25	3.67	4.08	10.48	11.54	8.79	1.88	3.31
τ_{00} (hospital)	2.35	1.07	1.36	6.70	1.47	2.95	1.05	1.08
ICC	0.25	0.23	0.25	0.39	0.11	0.25	0.36	0.25
**B. Quinolones use**								
Intercept	3.26 (1.76–4.77) [<0.001]	0.60 (-0.57–1.78) [0.322]	1.23 (0.05–2.43) [0.043]	4.01 (1.90–6.07) [<0.001]	1.49 (-0.45–3.40) [0.130]	3.09 (1.37–4.79) [0.001]	3.03 (2.15–3.91) [<0.001]	-2.39 (-4.19–-0.61) [0.007]
Time [year]	-0.57 (-0.82–-0.31) [<0.001]	0.22 (0.02–0.42) [0.032]	0.14 (-0.07–0.35) [0.182]	-0.11 (-0.45–0.23) [0.499]	0.25 (-0.09–0.59) [0.153]	-0.19 (-0.49–0.11) [0.218]	-0.50 (-0.65–-0.35) [<0.001]	0.61 (0.32–0.90) [<0.001]
Quinolones use in DDD per 1,000 hospital bed-days	0.32 (0.21–0.43) [<0.001]	0.11 (0.02–0.20) [0.014]	0.15 (0.06–0.24) [0.002]	0.25 (0.10–0.40) [0.002]	0.20 (0.05–0.34) [0.008]	0.24 (0.11–0.37) [0.001]	-0.07 (-0.14–-0.01) [0.028]	0.18 (0.04–0.32) [0.009]
σ^2	6.25	3.48	3.96	10.16	11.55	8.99	1.82	3.23
τ_{00} (hospital)	1.82	1.27	1.17	5.92	1.11	1.59	1.11	0.77
ICC	0.23	0.27	0.23	0.37	0.09	0.15	0.38	0.19
**C. Carbapenems use**								
Intercept	3.98 (2.13–5.86) [<0.001]	-0.45 (-1.70–0.77) [0.484]	1.66 (0.26–3.05) [0.026]	3.01 (0.40–5.54) [0.024]	-1.07 (-3.24–1.02) [0.322]	1.45 (-0.49–3.31) [0.148]	1.55 (0.53–2.59) [0.004]	-2.88 (-4.67–-1.11) [0.002]
Time [year]	-0.71 (-0.96–-0.45) [0.000]	0.26 (0.08–0.44) [0.003]	0.08 (-0.12–0.28) [0.423]	-0.07 (-0.39–0.26) [0.681]	0.42 (0.11–0.70) [0.005]	-0.09 (-0.37–0.19) [0.542]	-0.39 (-0.53–-0.24) [0.000]	0.60 (0.34–0.87) [0.000]
Carbapenem use in DDD per 1,000 hospital bed-days	0.09 (0.03–0.15) [0.006]	0.08 (0.04–0.12) [<0.001]	0.03 (-0.01–0.08) [0.114]	0.11 (0.04–0.19) [0.004]	0.16 (0.09–0.22) [<0.001]	0.14 (0.08–0.20) [<0.001]	0.03 (0.00–0.07) [0.032]	0.08 (0.03–0.13) [<0.001]
σ^2	6.41	3.19	3.95	9.41	9.24	7.69	1.84	2.79
τ_{00} (hospital)	3.60	1.01	1.43	7.26	1.96	2.37	1.15	1.22
ICC	0.36	0.24	0.27	0.44	0.18	0.24	0.39	0.30
Observations	243	242	237	237	242	243	242	166

Notes: Estimates were not fully adjusted using fixed effect variables, but only time and antibiotic use. DDD= Daily defined doses. ICC= Intraclass correlation. ICU= Intensive care unit. Coeff= Coefficient. CI= Confidence intervals. CPE= Carbapenemase-producing Enterobacterales. CRAB=Carbapenem-resistant *Acinetobacter baumannii*. CRE= Carbapenem-resistant Enterobacterales. CRPA= Carbapenem-resistant *Pseudomonas aeruginosa*. ESBL ECO= Extended spectrum beta-lactamase producing *Escherichia coli*. ESBL KPN= Extended spectrum beta-lactamase producing *Klebsiella pneumoniae.* MRSA= Methicillin-resistant *Staphylococcus aureus.* VRE= Vancomycin-resistant *Enterococcus* species.

Table S11. Results from Bayesian hierarchical models assessing antibiotic use as the main determinant of multidrug-resistant infection incidence in ICU settings, 2015-2024, by bug-drug combination

Variable	**MRSA** Coeff. (95%CI) [*p*]	**VRE** Coeff. (95%CI) [*p*]	**ESBL EC** Coeff. (95%CI) [*p*]	**ESBL KPN** Coeff. (95%CI) [*p*]	**CRE** Coeff. (95%CI) [*p*]	**CRPA** Coeff. (95%CI) [*p*]	**CRAB** Coeff. (95%CI) [*p*]	**CPE** Coeff. (95%CI) [*p*]
Intercept	3.31 (-1.37–7.77) [0.151]	1.55 (-0.65–3.71) [0.157]	3.46 (0.72–6.07) [0.018]	5.66 (1.61–9.76) [0.007]	0.06 (-3.16–3.40) [0.985]	2.80 (-1.81–7.31) [0.216]	0.93 (-1.96–3.85) [0.527]	0.23 (-2.05–2.44) [0.828]
Time [year]	-0.31 (-1.01–0.37) [0.379]	0.19 (-0.18–0.58) [0.330]	0.11 (-0.30–0.50) [0.581]	0.65 (0.01–1.28) [0.046]	0.28 (-0.27–0.81) [0.293]	0.37 (-0.42–1.14) [0.349]	-0.28 (-0.61–0.05) [0.092]	0.22 (-0.26–0.71) [0.349]
Carbapenem use in DDD per 1,000 hospital bed-days	0.08 (0.00–0.16) [0.050]	0.06 (0.03–0.10) [0.001]	-0.01 (-0.05–0.04) [0.677]	0.05 (-0.02–0.13) [0.169]	0.11 (0.05–0.16) [<0.001]	0.09 (0.01–0.17) [0.032]	0.04 (-0.01–0.09) [0.113]	0.00 (-0.03–0.03) [0.941]
Cephalosporins use in DDD per 1,000 hospital bed-days	0.00 (-0.09–0.10) [0.927]	-0.04 (-0.08–0.01) [0.085]	-0.02 (-0.07–0.04) [0.561]	0.00 (-0.08–0.09) [0.942]	0.04 (-0.03–0.10) [0.232]	-0.02 (-0.12–0.07) [0.653]	0.04 (-0.01–0.10) [0.127]	-0.01 (-0.04–0.02) [0.644]
Quinolones use in DDD per 1,000 hospital bed-days	0.29 (0.14–0.44) [<0.001]	0.04 (-0.03–0.11) [0.284]	0.06 (-0.02–0.15) [0.115]	0.06 (-0.07–0.20) [0.338]	0.05 (-0.06–0.16) [0.347]	0.15 (-0.01–0.30) [0.059]	-0.05 (-0.14–0.04) [0.269]	0.01 (-0.06–0.08) [0.720]
FE variables⁑	✓	✓	✓	✓	✓	✓	✓	✓
**Random effects**								
σ^2	5.58	1.88	1.90	4.92	3.79	8.43	0.99	0.33
τ_{00} (hospital)	4.17	0.27	0.84	2.80	1.13	1.96	3.93	0.12
ICC	0.43	0.13	0.31	0.36	0.23	0.19	0.80	0.26
R^2 marginal / conditional	0.49 / 0.69	0.35 / 0.44	0.21 / 0.42	0.39 / 0.61	0.36 / 0.49	0.27 / 0.42	0.26 / 0.84	0.27 / 0.40
Observations	243	242	237	237	242	243	242	166

Notes: DDD= Daily defined doses. ICU= Intensive care unit. Coeff= Coefficient. CI= Confidence intervals. FE= Fixed effect. ⁑ FE variables indicate fixed effect variables such as paediatric ICU, private hospital, infectious disease hours, poverty. CPE= Carbapenemase-producing Enterobacterales. CRAB=Carbapenem-resistant *Acinetobacter baumannii*. CRE= Carbapenem-resistant Enterobacterales. CRPA= Carbapenem-resistant *Pseudomonas aeruginosa*. ESBL ECO= Extended spectrum beta-lactamase producing *Escherichia coli*. ESBL KPN= Extended spectrum beta-lactamase producing *Klebsiella pneumoniae.* MRSA= Methicillin-resistant *Staphylococcus aureus.* VRE= Vancomycin-resistant *Enterococcus* species.

Figure S10. Key AMR and policy IPC milestones in Chile over recent years

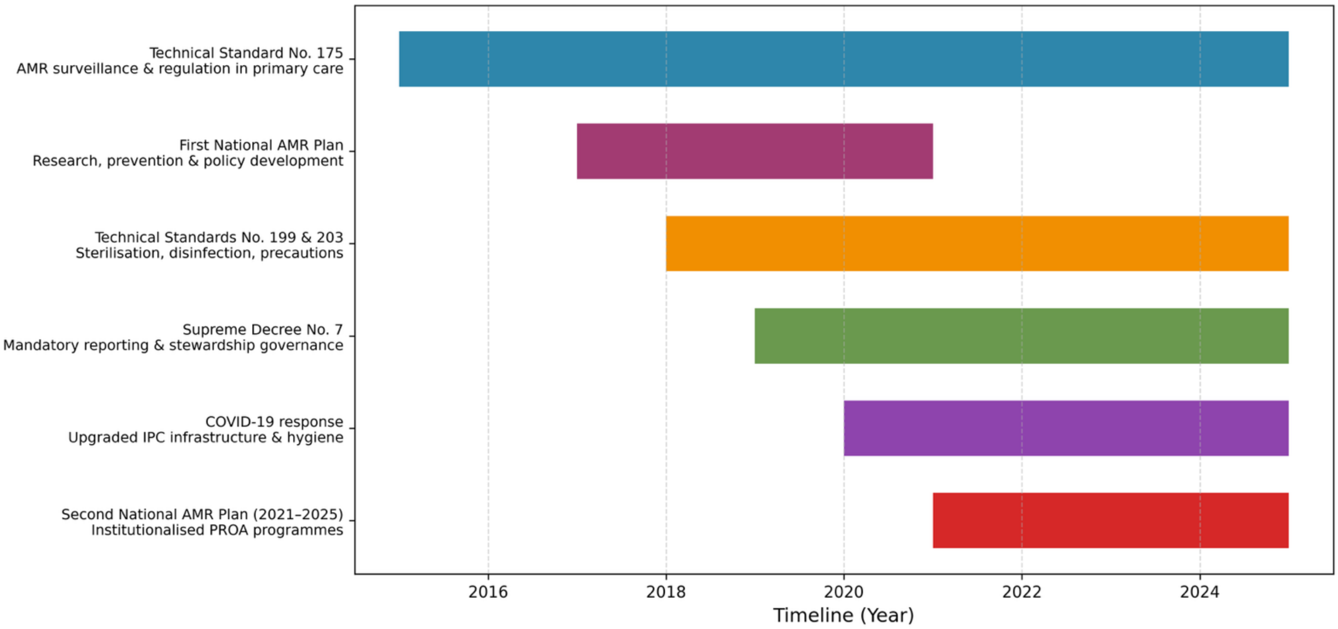

Notes: IPC= Infection prevention and control. AMR= Antimicrobial resistance. A national programme of IPC, launched in 1983, had already incorporated the principles of surveillance and prevention into hospital practice. However, since 2015, the system has been reinforced: the compulsory implementation of AMR surveillance has improved the awareness of resistant isolates, and the updated guidelines for sterilization, disinfection, and contact precautions have standardised the daily routines of ICUs. A change of approach, from recommendation to enforcement, has been highlighted by the second National AMR Plan. In 2019, a decree has established the mandatory reporting of resistant microorganisms and the governance of antimicrobial stewardship. In 2020, the implementation of the PROA programme has been mandatory in all hospitals, and the principles of prescribing oversight, audit, and feedback have been incorporated into the daily routines of ICUs. During the COVID pandemic, additional investments have been made in the infrastructure of IPC and diagnostic tools to enhance the isolation capabilities and the microbiological detection of ICUs.

STROBE Guidelines

STROBE Statement—checklist of items that should be included in reports of observational studies

	Item No	Recommendation
**Title and abstract**	1	(*a*) Indicate the study's design with a commonly used term in the title or the abstract: **This was included, see title, first page.**
		(*b*) Provide in the abstract an informative and balanced summary of what was done and what was found. **This was included, see title, first page.**
**Introduction**		
Background/rationale	2	Explain the scientific background and rationale for the investigation being reported. **See introduction, page 4.**
Objectives	3	State specific objectives, including any prespecified hypotheses. **See introduction, page 4.**
**Methods**		
Study design	4	Present key elements of study design early in the paper. **See Methods, Study design and population subsection.**
Setting	5	Describe the setting, locations, and relevant dates, including periods of recruitment, exposure, follow-up, and data collection **See Methods, Study design and population subsection.**
Participants	6	(*a*) *Cohort study*—Give the eligibility criteria, and the sources and methods of selection of participants. Describe methods of follow-up *Case-control study*—Give the eligibility criteria, and the sources and methods of case ascertainment and control selection. Give the rationale for the choice of cases and controls *Cross-sectional study*—Give the eligibility criteria, and the sources and methods of selection of participants **See Methods, Study design and population subsection.**
		(*b*) *Cohort study*—For matched studies, give matching criteria and number of exposed and unexposed *Case-control study*—For matched studies, give matching criteria and the number of controls per case
Variables	7	Clearly define all outcomes, exposures, predictors, potential confounders, and effect modifiers. Give diagnostic criteria, if applicable **See Methods, Incidence density rate AND independent variables subsections.**
Data sources/ measurement	8*	For each variable of interest, give sources of data and details of methods of assessment (measurement). Describe comparability of assessment methods if there is more than one group. **See Methods, Incidence density rate AND independent variables subsections.**
Bias	9	Describe any efforts to address potential sources of bias. **See Methods, Statistical analyses subsection.**
Study size	10	Explain how the study size was arrived at. **See Methods, Statistical analyses subsection.**
Quantitative variables	11	Explain how quantitative variables were handled in the analyses. If applicable, describe which groupings were chosen and why. **See Methods, Statistical analyses subsection.**
Statistical methods	12	(*a*) Describe all statistical methods, including those used to control for confounding. **See Methods, Statistical analyses subsection.**
		(*b*) Describe any methods used to examine subgroups and interactions. **See Methods, Statistical analyses subsection.**
		(*c*) Explain how missing data were addressed. **See Methods, Statistical analyses subsection.**

		(*d*) *Cohort study*—If applicable, explain how loss to follow-up was addressed<br>*Case-control study*—If applicable, explain how matching of cases and controls was addressed<br>*Cross-sectional study*—If applicable, describe analytical methods taking account of sampling strategy
		(*e*) Describe any sensitivity analyses. **See Methods, Statistical analyses subsection.**
**Results**		
Participants	13*	(a) Report numbers of individuals at each stage of study—eg numbers potentially eligible, examined for eligibility, confirmed eligible, included in the study, completing follow-up, and analysed. **See Results, Descriptive statistics of the included ICUs subsection.**
		(b) Give reasons for non-participation at each stage
		(c) Consider use of a flow diagram **See Results, Descriptive statistics of the included ICUs subsection; and Methods.**
Descriptive data	14*	(a) Give characteristics of study participants (eg demographic, clinical, social) and information on exposures and potential confounders. **See Results, Descriptive statistics of the included ICUs subsection.**
		(b) Indicate number of participants with missing data for each variable of interest. **See Results, Descriptive statistics of the included ICUs subsection.**
		(c) *Cohort study*—Summarise follow-up time (eg, average and total amount)
Outcome data	15*	*Cohort study*—Report numbers of outcome events or summary measures over time. **See Results, Descriptive statistics of the included ICUs subsection; and Variability in IDRs and ABU over time subsection.**
		Case-control study—Report numbers in each exposure category, or summary. measures of exposure.
		Cross-sectional study—Report numbers of outcome events or summary measures
Main results	16	(*a*) Give unadjusted estimates and, if applicable, confounder-adjusted estimates and their precision (eg, 95% confidence interval). Make clear which confounders were adjusted for and why they were included. **See Results, Sociodemographic and hospital determinants of MDR IDRs subsection, and Antibiotic use as determinant of MDR and drug-bug combination IDRs section.**
		(*b*) Report category boundaries when continuous variables were categorized
		(*c*) If relevant, consider translating estimates of relative risk into absolute risk for a meaningful time period
Other analyses	17	Report other analyses done—eg analyses of subgroups and interactions, and sensitivity analyses. **See Results, Sociodemographic and hospital determinants of MDR IDRs subsection, and Antibiotic use as determinant of MDR and drug-bug combination IDRs section.**
**Discussion**		
Key results	18	Summarise key results with reference to study objectives. **See Discussion, first paragraph.**
Limitations	19	Discuss limitations of the study, taking into account sources of potential bias or imprecision. Discuss both direction and magnitude of any potential bias. **See Discussion, limitations paragraph.**
Interpretation	20	Give a cautious overall interpretation of results considering objectives, limitations, multiplicity of analyses, results from similar studies, and other relevant evidence. **See Discussion.**
Generalisability	21	Discuss the generalisability (external validity) of the study results. **See Discussion,**
**Other information**		

Funding	22	Give the source of funding and the role of the funders for the present study and, if applicable, for the original study on which the present article is based. **See first page under the abstract.**

*Give information separately for cases and controls in case-control studies and, if applicable, for exposed and unexposed groups in cohort and cross-sectional studies.

Note: An Explanation and Elaboration article discusses each checklist item and gives methodological background and published examples of transparent reporting. The STROBE checklist is best used in conjunction with this article (freely available on the Web sites of PLoS Medicine at http://www.plosmedicine.org/, Annals of Internal Medicine at http://www.annals.org/, and Epidemiology at http://www.epidem.com/). Information on the STROBE Initiative is available at www.strobe-statement.org.
